# Cyanophages from a less virulent clade dominate over their sister clade in global oceans

**DOI:** 10.1038/s41396-022-01259-y

**Published:** 2022-06-20

**Authors:** Ilia Maidanik, Shay Kirzner, Irena Pekarski, Laure Arsenieff, Ran Tahan, Michael C. G. Carlson, Dror Shitrit, Nava Baran, Svetlana Goldin, Joshua S. Weitz, Debbie Lindell

**Affiliations:** 1 Faculty of Biology, Technion—Israel Institute of Technology, Haifa, 3200003 Israel; 2 School of Biological Sciences, Georgia Institute of Technology, Atlanta, GA 30332 USA; 3 School of Physics, Georgia Institute of Technology, Atlanta, GA 30332 USA; 4 Institut de Biologie, École Normale Supérieure, Paris, 75014 France

**Keywords:** Microbial ecology, Molecular ecology, Bacteriophages, Population dynamics, Microbial biooceanography

## Abstract

Environmental virus communities are highly diverse. However, the infection physiology underlying the evolution of diverse phage lineages and their ecological consequences are largely unknown. T7-like cyanophages are abundant in nature and infect the marine unicellular cyanobacteria, *Synechococcus* and *Prochlorococcus*, important primary producers in the oceans. Viruses belonging to this genus are divided into two distinct phylogenetic clades: clade A and clade B. These viruses have narrow host-ranges with clade A phages primarily infecting *Synechococcus* genotypes, while clade B phages are more diverse and can infect either *Synechococcus* or *Prochlorococcus* genotypes. Here we investigated infection properties (life history traits) and environmental abundances of these two clades of T7-like cyanophages. We show that clade A cyanophages have more rapid infection dynamics, larger burst sizes and greater virulence than clade B cyanophages. However, clade B cyanophages were at least 10-fold more abundant in all seasons, and infected more cyanobacteria, than clade A cyanophages in the Red Sea. Models predicted that steady-state cyanophage abundances, infection frequency, and virus-induced mortality, peak at intermediate virulence values. Our findings indicate that differences in infection properties are reflected in virus phylogeny at the clade level. They further indicate that infection properties, together with differences in subclade diversity and host repertoire, have important ecological consequences with the less aggressive, more diverse virus clade having greater ecological impacts.

## Introduction

Bacteriophages are considered major effectors in microbial ecology. They influence the abundance, diversity, and evolution of their hosts [1, 2]. They also impact global biogeochemical cycles through the lysis of host cells and the release of cellular material into the water column [1, 3]. Bacteriophages themselves are highly diverse, with many different phage subfamilies and genera [4, 5]. Within genus diversity is manifested both by allelic diversity of core genes present in all members of the genus and gene content beyond the core [6]. This diversity is largely the result of low replication fidelity and high levels of recombination relative to cellular life [6–8]. Phylogenetic analysis of core genes has revealed discrete lineages within phage genera [9, 10]. Recently, some of these discrete lineages were found to form distinct genetic populations in nature [6, 11, 12] and may thus be the result of adaptive processes [6]. However, the biology underlying the evolution of discrete phage lineages remains largely unknown. Of particular interest is whether phage phylogeny mirrors differences in life history traits (phage physiology and infection properties) and how such variability influences phage ecology (abundances and distribution patterns) and their ecological impacts (host infection and mortality).

One of the most well-studied groups of environmental phages are the cyanophages, particularly those infecting the marine unicellular cyanobacteria belonging to the genera *Synechococcus* and *Prochlorococcus* [6, 9, 13–16]. These two cyanobacterial genera are made up of a diverse set of organisms that form discrete ecotypes [17, 18]. Collectively they are the most abundant photosynthetic organisms on Earth and are responsible for a significant fraction of global primary production [19]. *Synechococcus* and *Prochlorococcus* have overlapping habitats, yet display distinct differences in their distribution patterns that are exhibited across large geographic expanses as well as seasonally, and correlate with changes in physical and chemical water column conditions [20–22]. Predation by planktonic grazers [23] and infection and lysis by cyanophages [1, 24–26] are also thought to be important factors influencing cyanobacterial diversity and abundance.

The marine cyanophages are tailed double-stranded DNA-containing phages belonging to the order *Caudovirales*. They are taxonomically separated into three families based on tail morphology: the *Myoviridae, Podoviridae* and *Siphoviridae*, and to subfamilies and genera based on replication strategies, genome characteristics and host range [4]. This study focuses on a genus within the *Podoviridae*, the T7-like cyanopodoviruses, which we refer to here as T7-like cyanophages. The T7-like cyanophages are lytic [27, 28] and are characterized by a narrow host range [10, 15]. Members of this genus cluster phylogenetically into two well defined lineages, clade A and clade B, based on phylogeny of both single genes (DNA polymerase and major capsid protein) and concatenated core genes [10, 16, 29]. Some differences between the clades are known. First, most clade A cyanophages infect a member of the *Synechococcus* genus with only a single isolate known to infect *Prochlorococcus*, while clade B phages infect either a *Synechococcus* or a *Prochlorococcus* strain [10]. Second, despite a conserved genomic core of homologous DNA replication and morphogenesis genes [16, 29], members of these clades differ in the presence of some non-core genes of (cyano)bacterial origin, termed auxiliary metabolic genes [10, 13, 16]. For example, the photosynthesis-related genes, *psbA* and *hli*, are encoded by nearly all clade B phages, but are absent from clade A phages, while most clade A phages code for a thioredoxin gene which is absent from clade B phages. Third, there is a greater degree of genetic diversity among clade B phages, with more subclades than among the clade A phages, as seen from greater allelic diversity of their core genes [13, 14]. Despite these distinctions, it is unknown whether the phylogenetic separation into clade A and clade B phages reflects differences in infection physiology. Furthermore, it is unclear how infection physiology, in turn, influences cyanophage population dynamics and infection patterns over changing environmental conditions in the oceans.

In this study, we investigated the infection properties of diverse clade A and clade B T7-like cyanophages under controlled laboratory conditions. We found that clade A cyanophages were more virulent, had a more rapid infection cycle and produced more phage progeny than clade B cyanophages. In addition, analysis of their annual population dynamics in the Gulf of Aqaba, Red Sea, showed that clade B T7-like cyanophages were more abundant than clade A cyanophages by at least an order of magnitude in all seasons and throughout the photic zone, and that they infected more cells. These findings revealed distinct differences in infection physiology and environmental abundances that mirror phage phylogeny, indicating that the separation into discrete lineages was likely a result of adaptation. Furthermore, modeling of these results suggest that the less aggressive infection strategy can maintain host populations at higher densities that ultimately support larger phage populations.

## Results and discussion

### Infection properties of clade A and clade B T7-like cyanophages

We set out to test the hypothesis that the phylogenetic separation of T7-like cyanophages into two major clades reflects differences in their infection physiology. To do this we investigated a suite of infection properties of three pairs of clade A and B phages, each pair infecting the same *Synechococcus* host (Table 1) to allow us to control for variability in host genetics and physiology. These six cyanophages are representatives of 3 clade A and 2 clade B cyanophage subclades (SI Appendix, Table S1).

**Table 1:** Summary of infection physiology of three pairs of clade A and clade B cyanophages infecting the same *Synechococcus* hosts.

Host and virus pairs			Adsorption kinetics		Latent period		Burst size		Virulence		Decay	
Host	Virus	Clade	Min^−1^ mean ± SE	Statistics Multi-level modeling	h mean	Statistics Multi-level modeling^a^	Phage∙cell^−1^ mean ± SD	Statistics Student t.test	% of lysed cells mean ± SD	Statistics Student *t*.test	ln (phage∙ml^−1^ day^−1^) mean±c.i.95%	Statistics Multi-level modeling
WH8109	Syn5	A	0.12 ± 0.016^b^ *n*_exp_ = 3 *n*_points_ = 18	Df = 22.98 *t* = −7.079 *p* = 3.28e−07	1 *n*_exp_ = 3 *n*_points_ = 21	Df = 146.8 *t* = −12.25 *p* = 2e−16	94.1 ± 61.7 *n*_*e*xps_ = 5 *n*_cells_ = 270	Df = 270.26 *t* = 24.5 *p* = 2.2e−16	75.8 ± 15.3 *n*_exps_ = 21	Df = 5.403 *t* = 6.407 *p* = 0.001	0.019 ± 0.005 *n*_exp_ = 3 *n*_points_ = 42	Df = 76.15 *t* = 1.34 *p* = 0.183
	S-TIP37	B	0.018 ± 0.002^b^ *n*_exp_ = 3 *n*_points_ = 18		3 *n*_exp_ = 3 *n*_points_ = 21		11.5 ± 11.7 *n*_exps_ = 10 *n*_cells_ = 221		14.3 ± 9.0 *n*_exps_ = 21		0.025 ± 0.007 *n*_exp_ = 3 *n*_points_ = 42	
WH7803	S-CBP42	A	0.022 ± 0.003 *n*_exp_ = 8 *n*_points_ = 40	Df = 71.01 *t* = −9.671 *p* = 1.37e−14	3 *n*_exp_ = 8 *n*_points_ = 84	Df = 38 *t* = 2.9 *p* = 0.006	95.25 ± 49.35 *n*_exps_ = 5 *n*_cells_ = 125	Df = 63.8 *t* = 4.311 *p* = 5.741e−05	41.7 ± 4.46 *n*_exps_ = 8	Df = 1.96 *t* = 5.6 *p* = 0.03	0.024 ± 0.006 *n*_exp_ = 3 *n*_points_ = 42	Df = 64 *t* = −1.528 *p* = 0.132
	S-RIP2	B	0.002 ± 0.0003 *n*_exp_ = 6 *n*_points_ = 47		8 *n*_exp_ = 6 *n*_points_ = 78		53.5 ± 55.6 *n*_exps_ = 5 *n*_cells_ = 42		19.2 ± 4.7 *n*_exps_ = 6		0.034 ± 0.012 *n*_exp_ = 3 *n*_points_ = 70	
CC9605	S-TIP28	A	0.031 ± 0.0007 *n*_exp_ = 4 *n*_points_ = 8	not determined^c^	2.5 *n*_exp_ = 4 *n*_points_ = 40	Df = 76 *t* = −10.23 *p* = 6.1e−16	122 ± 95.5 *n*_exps_ = 5 *n*_cells_ = 166	Df = 283.32 *t* = 8.864 *p* = 2.2e−16	57.9 ± 6.47 *n*_exps_ = 8	Df = 5.102 *t* = −0.976 *p* = 0.373	0.011 ± 0.009 *n*_exp_ = 3 *n*_points_ = 42	Df = 76 *t* = −3.877 *p* = 0.03
	S-TIP67	B	0.002 ± 0.0003 *n*_exp_ = 4 *n*_points_ = 24		12 *n*_exp_ = 4 *n*_points_ = 40		46.6 ± 55.3 *n*_exps_ = 5 *n*_cells_ = 121		56.7 ± 4.51 *n*_exps_ = 12		−0.0004 ± 0.006 *n*_exp_ = 3 *n*_points_ = 42	

*n*_exp_ relates to the number of independent experiments, *n*_points_ relates to the number of points in the combined number of experiments, *n*_cells_ relates to the number of cells analyzed.

^a^Statistics of overall infection dynamics.

^b^Experiments done at MOI = 0.01.

^c^The adsorption for these two phages was measured with different experimental designs (see Methods) so they cannot be compared statistically.

**Fig. 1:**
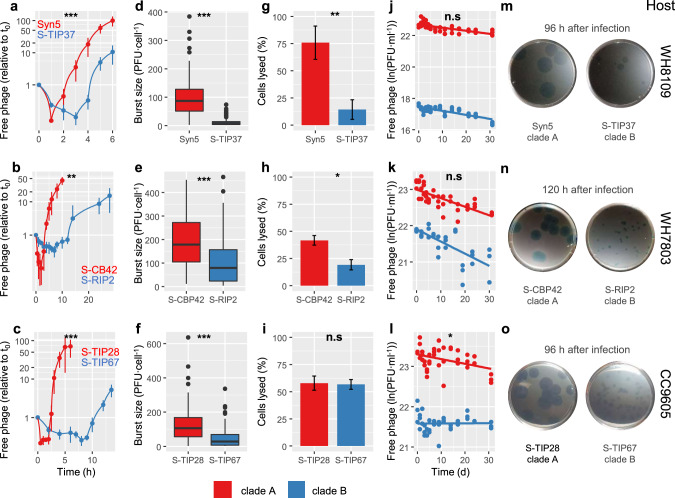
Comparison of the infection physiology between pairs of clade A and clade B T7-like cyanophage infecting the same *Synechococcus* host. **a**–**c** Cyanophage growth curves, **d**–**f** burst sizes, **g**–**i** virulence as the percentage of lysed host cells, **j**–**l** decay as loss of infectivity, **m**–**o** plaque sizes. **a**, **d**, **g**, **j**, **m** Clade A Syn5 phage and clade B S-TIP37 phage infecting WH8109. **b**, **e**, **h**, **k**, **n** Clade A S-CBP42 phage and clade B S-RIP2 phage infecting WH7803. **c**, **f**, **i**, **l**, **o** Clade A S-TIP28 phage and clade B S-TIP67 phage infecting CC9605. The host strain is shown at the right of the panels. Red and blue lines or bars show results for clade A and clade B phages, respectively. **a**–**c**, **g**–**I** Error bars indicate standard deviations. **d**–**f** Burst size results are for single cells. **j**–**l** The solid line shows the fitted multi-level linear model. **m**–**o** The time after infection at which plaques were photographed appears above the images. *p value < 0.05; **p value < 0.01; ***p value < 0.001; n.s. *p* > 0.05. Means, variances, number of replicates and *p* values are shown in Tables 1 and S3.

We began by investigating adsorption kinetics and the length of time taken to produce new phages in the infection cycle, the latent period, from phage growth curve experiments. In all three pairs of phages, adsorption was 7–15-fold more rapid in the clade A phage versus the clade B phage (Fig. 1, Table 1). Furthermore, the clade A phage had a faster infection cycle with a latent period that was 3-5-fold shorter than the clade B phage on the same host (Fig. 1a–c) (Table 1). To determine how representative these findings are for a greater diversity of T7-like cyanophages we report the latent period of nine additional non-paired phages that infect a variety of hosts and span the diversity of this cyanophage genus, measured here and taken from the literature (SI Appendix, Table S1). These phages showed the same pattern as observed between phage pairs, although one clade A phage had a relatively long latent period (see SI Appendix, Table S1). Overall, the 5 clade A phages representative of 5 subclades had a significantly shorter latent period (3.3 ± 3.6 h, *n* = 5 phages (mean ± SD) than the 10 clade B phages from 7 subclades (7.7 ± 2.0 h, *n* = 10 phages) (Kruskal-Wallis: *χ*^2^ = 4.72, df = 1; *p* = 0.029, *n* = 15). No significant differences in the length of the latent period were found for clade B phages that infected *Synechococcus* and *Prochlorococcus* (Kruskal-Wallis: *χ*2 = 1.13, df = 1; *p* = 0.29, *n* = 10).

We determined the number of infective phage progeny produced per cell, the burst size, using a single cell approach [30]. In this assay, single infected cells are separated by flow cytometry into individual wells at maximal adsorption (SI Appendix, Table S2), allowed to lyse, and the number of infective phages produced is determined by the plaque assay (see Methods). The three clade A phages had significantly larger burst sizes (135.5 ± 49.6 phages·cell^−1^) than the three clade B phages (55.0 ± 48.2 phages·cell^−1^) (paired *t*-test: df = 2, *t* = 5.28, *p* = 0.03, *n* = 6) by 2-8-fold for each phage pair, as determined from 42–270 individual cells in five independent experiments (Fig. 1d–f, Table 1). Thus, clade A phages had higher burst sizes relative to clade B phages despite having a shorter infection cycle. This finding challenges current thinking that the evolution of shorter latent periods necessarily results in a tradeoff of smaller burst sizes for all phage types [31, 32].

The combined effects of all stages of infection, including adsorption kinetics, the length of the latent period and the burst size can be seen from the timing of plaque formation and their size [33]. Clade A plaques became visible in less than 24 h post infection while clade B plaques took 3-4 days to appear. Furthermore, 2.4–4.3-fold larger plaques were produced by clade A phages (11–16 mm) than clade B phages (3–7 mm) over the same period of time (paired *t*-test: df = 2, *t* = 13.714, *p* = 0.0052, *n* = 6) (Fig. 1m–o) (SI Appendix, Table S3). These findings show that, under these laboratory conditions, clade A phages have significantly greater fitness than clade B phages on the same hosts.

We then quantified virulence which we define here as the probability that a phage kills and lyses a cell after adsorption [30, 34]. This was determined from the percentage of individual cells in a population that were lysed by each phage using a single cell approach [30]. The host was challenged with the same number of infective phages at a multiplicity of infection (MOI) of 2, and each phage was allowed to adsorb to the host until maximal adsorption was achieved (see Methods and SI Appendix, Table S2). The clade A phage lysed between a 2–3-fold higher proportion of cells than the clade B phage when comparing two of the phage pairs, whereas no significant difference was found for the third phage pair (Fig. 1g–i, Table 1).

The viability of a phage is affected by its extracellular decay rate which influences the period of time it has to encounter and infect a new host. To assess whether rates of decay differ between clade A and clade B phages we determined the loss of infectious phages over time when incubated under host growth conditions. No consistent pattern was observed across phage pairs (Fig. 1j–l, Table 1) and there was no significant difference in mean decay rates between clade A and clade B phages (*t*-test: df = 0.13, *t* = 2.5308, *p* = 0.9, *n* = 6).

These combined findings show that for most infection properties, clade A phages are more aggressive than clade B phages as they complete their infection cycle more rapidly, produce more progeny and kill more host cells. Thus, phylogenetic differences within a diverse, ecologically important phage genus have clear manifestations in infection properties at the clade level and are thus likely to be a result of adaptation and selection. These differences in infection properties are present even though members of both clades have the same genomic backbone of replication and morphology genes and infect the same cyanobacterial host taxa. The genomic underpinnings of the observed clade-level differences in infection properties are currently unknown and could be due to allelic differences in core genes, the result of distinct gene repertoires, or a combination of both.

### Annual population dynamics of T7-like cyanophages in the Gulf of Aqaba

Differences in infection properties are expected to influence the abundance and distribution patterns of cyanophages. We assessed the population dynamics of clade A and clade B cyanophages over the annual cycle in the Gulf of Aqaba, Red Sea. To do so, we collected samples from monthly depth profiles over a 1-year period, focusing on the upper 140 m photic zone where their cyanobacterial hosts reside. To put our findings into their environmental context, we first describe the seasonal dynamics of the water column and cyanobacterial distributions in these waters.

#### Physicochemical conditions of the water column and cyanobacterial population dynamics

**Fig. 2:**
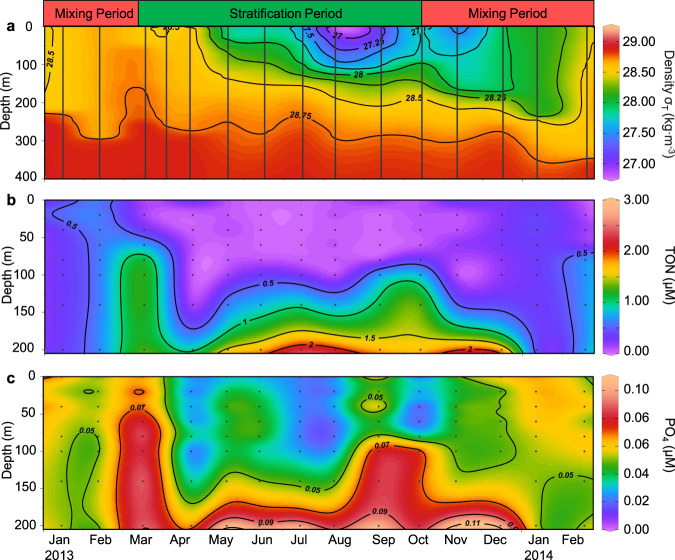
The annual dynamics of water column density and nutrients at Station A in the Gulf of Aqaba, Red Sea. **a** σ density anomaly (**b**) Total oxidized nitrogen, NO_2_^−^ + NO_3_^−^ (TON), **c** phosphate (PO_4_). The contours are interpolations performed using DIVA gridding. All plots were created by Ocean Data View (http://odv.awi.de/). The data from 4 April 2013 were published previously [37] and are shown here for complete presentation of the annual cycle.

**Fig. 3:**
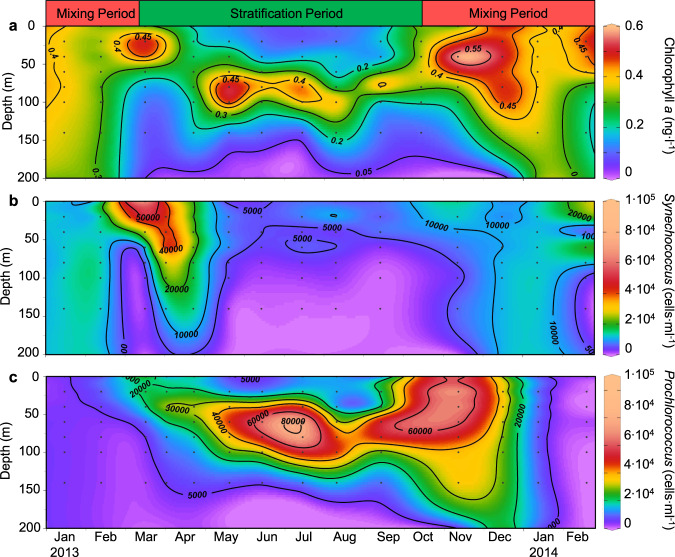
The annual dynamics of phytoplankton at Station A in the Gulf of Aqaba, Red Sea. **a** Extracted chlorophyll *a* concentrations, **b** *Synechococcus* abundances, **c** *Prochlorococcus* abundances. Black points indicate samples. The contours are interpolations performed using DIVA gridding. All plots were created by Ocean Data View (http://odv.awi.de/). The data from 4 April 2013 were published previously [37] and are shown here for complete presentation of the annual cycle.

The Gulf of Aqaba has a characteristic seasonal cycle in water column stability (see Methods), affecting nutrient availability and phytoplankton abundances in the photic zone [20, 35] that was also observed over our period of sampling (Figs. 2, 3 and SI Appendix, Fig. S1). Winter mixing reached a depth of ~300 m by the end of February 2013 (Fig. 2a) and injected nutrients into the photic zone (Fig. 2b, c). Stratification began upon warming of the upper surface layers in March. Maximal stratification was observed by August. During the stratification period nutrients in the photic zone were utilized by the phytoplankton and dropped below limits of detection by mid-spring (Fig. 2b, c). Mixing commenced again in October as the upper layers cooled. The mixed layer extended below the photic zone by the middle of December and reached its maximal depth again in February 2014 (Fig. 2a). For simplicity we refer to two periods that differ in their water column stability: the stratification period from March to September and the mixing period from October to February.

Chlorophyll *a*, present in all primary producers, is often used as an approximate proxy for phytoplankton biomass. Chlorophyll *a* concentrations and phytoplankton group abundances were uniformly distributed throughout the mixed layer during periods of mixing (Fig. 3, and SI Appendix, Fig. S1a). A shallow subsurface peak in chlorophyll *a* concentration (the deep chlorophyll maximum, DCM) developed early during stratification in the spring and deepened to 100 m by the summer (Fig. 3a). The DCM coincided with maximal abundances of small photosynthetic eukaryotes and *Synechococcus* during the short-lived spring bloom and with *Prochlorococcus* and photosynthetic eukaryotes in summer (Fig. 3, and SI Appendix, Fig. S1a). As found previously, eukaryotic phytoplankton, *Synechococcus* and *Prochlorococcus* were most abundant in winter, spring and summer, respectively [20].

#### T7-like cyanophage annual population dynamics

**Fig. 4:**
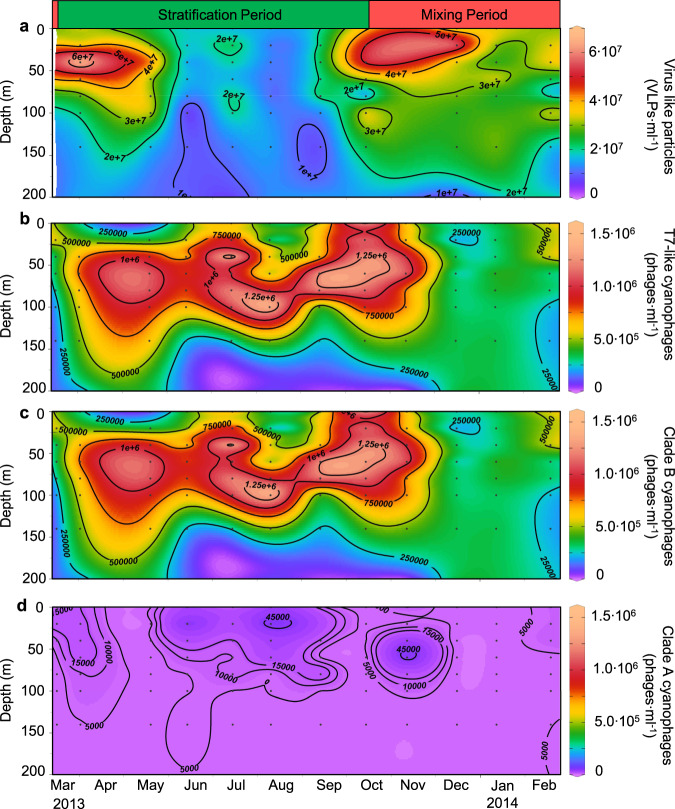
The annual dynamics of viruses at Station A in the Gulf of Aqaba, Red Sea. **a** Virus-like particle abundances, **b** total T7-like cyanophage abundances, **c** clade B T7-like cyanophage abundances, **d** clade A T7-like cyanophage abundances. Black points indicate samples. The contours are interpolations performed using DIVA gridding. All plots were created by Ocean Data View (http://odv.awi.de/). See SI Appendix, Fig. S3 for individual depth profiles of T7-like cyanophages. The data from 4 April 2013 in (**b**–**d**) were published previously [37] and are shown here for complete presentation of the annual cycle.

Virus abundances were determined from a total of 107 samples from 12 depth profiles collected from March 2013 to February 2014. First we quantified virioplankton from virus-like particles (VLPs) which are generally considered to reflect abundances of dsDNA viruses [36]. VLPs were most abundant in transition periods as the water column changed from mixing to stratification (March to May) and from stratification to mixing (October to December) (Fig. 4a). Maximal abundances of 5–7 × 10^7^ VLPs·ml^−1^ were observed in the upper 60 m of the water column in April and October. Abundances were lowest during stable stratification from June to September, but were still observed at densities in excess of 10^7^ VLPs·ml^−1^ (Fig. 4a). VLP abundances were significantly correlated with trophic status of the water column, represented by chlorophyll *a* concentration (ρ = 0.62, *p* = 1.1 × 10^−8^; *n* = 68) as well as with *Synechococcus* (ρ = 0.68 *p* = 1.6 × 10^−10^; *n* = 69) and heterotrophic bacteria (ρ = 0.28, *p* = 0.014; *n* = 75).

We quantified clade A and clade B T7-like cyanophages over the annual cycle in the Gulf of Aqaba using the polony method, a solid-phase single-molecule PCR method [37]. T7-like cyanophage population dynamics were quite different to those of total VLPs (Fig. 4a, b). Maximal abundances of T7-like cyanophages were observed during stable stratification when VLPs were at their annual minimum. Thus, while T7-like cyanophages made up between 0.3–12% of the VLPs over the annual cycle, they contributed most to the virioplankton pool between June-September, with a maximum contribution of 12.1 ± 6.8% of VLPs at 100 m in August (SI Appendix, Fig. S2). These findings show that T7-like cyanophages have different population dynamics compared to the dsDNA virus community as a whole.

T7-like cyanophage populations were dominated by clade B cyanophages (Fig. 4b–d, and SI Appendix, Fig. S3). Their maximal monthly abundances typically ranged from 0.6–1.5 × 10^6^ phage·ml^−1^. In contrast, clade A cyanophage abundances were never higher than 6.0 × 10^4^ phages·ml^−1^ and were below the limit of accurate quantification (1 × 10^4^ phages·ml^−1^) in 75% of the samples (Fig. 4d, and SI Appendix, Fig. S3). As such, clade B cyanophages were more abundant than clade A cyanophages at all depths and in all seasons at ratios that ranged from 2.8-fold to over a 1000-fold. In fact, clade B phages were at least an order of magnitude more abundant than clade A cyanophages in 97% of all samples collected in the photic zone (*n* = 84).

**Fig. 5:**
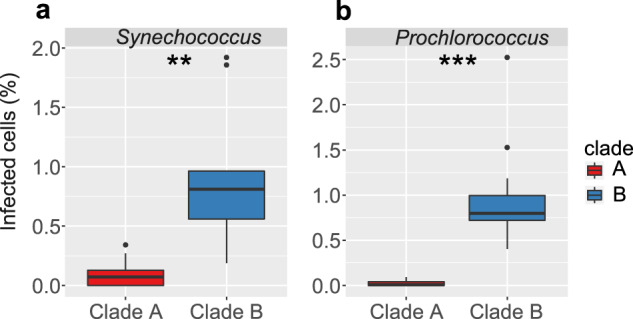
Proportion of cyanobacterial populations infected by T7-like cyanophages at Station A in the Gulf of Aqaba, Red Sea. **a** *Synechococcus* infection (*n* = 18), and **b** *Prochlorococcus* infection (*n* = 24), by clade A (red) and clade B (blue) T7-like cyanophages from samples collected in March and September 2014. **p value < 0.01; ***p value < 0.001.

We then assessed whether differences in environmental cyanophage abundances translated into differences in the extent of infection. We assessed the percent of infected *Synechococcus* and *Prochlorococcus* cells by clade A and clade B phages during March and September in 2014 using the iPolony method [38]. The less aggressive clade B cyanophages infected significantly more cyanobacteria than clade A phages in all but one sample (paired Wilcoxon test: *V* = 44, *p* = 0.0078, *n* = 18 for *Synechococcus* and *V* = 78, *p* = 0.0004, *n* = 24 for *Prochlorococcus*) (Fig. 5). Moreover, in 85% of the samples clade B phages infected at least 10-fold more cyanobacteria than clade A phages.

Distribution patterns of clade A and clade B cyanophages changed with seasonal shifts in water column conditions and cyanobacterial abundances. At the beginning of the stratification period in March 2013, clade B cyanophage abundances were highest in the upper 60 m (3.3 × 10^5^–5.5 × 10^5^ phages·ml^−1^), coincident with the *Synechococcus* bloom (Fig. 4b and SI Appendix, Fig. S3). Their numbers increased and the maximum deepened as stratification intensified during spring-summer, coinciding with the peak in *Prochlorococcus*. Annual maxima in clade B cyanophage abundances were observed in the summer with highest numbers in August at 100 m (1.58 × 10^6^ ± 0.63 × 10^6^ phages·ml^−1^) (mean ± ci95%). Abundances remained relatively high through the beginning of the autumn mixing period in October-November. As mixing progressed, abundances became uniformly distributed over the mixed layer and dropped down to 1.8 × 10^5^ phages·ml^−1^ (Fig. 4c, and SI Appendix, Fig. S3). Overall, clade B cyanophage abundances correlated with *Prochlorococcus* (assessed for 60 to 140 m depth, see Methods) (ρ = 0.83, *S* = 2378, *p* < 2.2 × 10^−16^, *n* = 44), especially during the stratification period (ρ = 0.93, *S* = 262, *p* < 2.2 × 10^−16^, *n* = 28). Since members of clade B cyanophages can infect either a *Synechococcus* or a *Prochlorococcus* host [10], the greater correlation with *Prochlorococcus* may be explained by their higher abundances relative to *Synechococcus* during stable stratification, supporting an overall larger population of clade B cyanophages.

Clade A cyanophages had somewhat similar seasonal dynamics to those of clade B cyanophages. They were most abundant during the stable stratification period (June-September) and during early mixing (November) (Fig. 4d). However, clade A cyanophages were present at notably shallower depths than clade B cyanophages, being most abundant in the upper 60 m of the water column throughout the stratification period (Fig. 4d, and SI Appendix, Fig. S3). Similar to clade B phages, maximal annual abundances of clade A cyanophages were found in August but at 20 m (5.8 × 10^4^ ± 2.0 × 10^4^ phages·ml^−1^) with similarly high abundances also found in November at 60 m (5.1 × 10^4^ ± 2.0 × 10^4^ phages·ml^−1^). Since clade A cyanophages were often close to or below the limit of accurate quantification, we concentrated samples from four depth profiles in different seasons (SI Appendix, Fig. S3). Correlation analysis with data from these profiles showed that clade A phages were highly correlated with *Synechococcus* during months of stratification (ρ = 0.93–0.97, *p* < 0.005, *n* = 7 per profile) but not during mixing (ρ = 0.12, *p* = 0.65, *n* = 7) (SI Appendix, Fig. S4). The correlation with *Synechococcus* rather than *Prochlorococcus* is expected given that cyanophages belonging to this clade primarily infect *Synechococcus* [10], (Fig. 5).

T7-like cyanophage dynamics correlated to seasonal changes in cyanobacterial populations. Such linked dynamics might be expected at the population level since phages are obligate intracellular parasites that require their hosts to replicate and because T7-like cyanophage dynamics were measured on monthly time scales that integrate dynamics across many cyanophage infection cycles (hours) and cyanobacterial divisions (on the order of a day). Previous findings investigating cyanophages over seasonal cycles at a similar temporal resolution, but at different taxonomic levels, show similar correlations between cyanobacterial and cyanophage abundances. These include studies that used infective assays measuring cyanophages that infect a specific cyanobacterium [39–41] and an amplicon study investigating single cyanobacterial and T4-cyanophage genotypes [42].

This study of T7-like cyanophage populations revealed the dominance of clade B over clade A cyanophages at all depths and in all seasons over the annual cycle in the Gulf of Aqaba, Red Sea. This dominance was apparent both when *Prochlorococcus* was the more abundant cyanobacterium in late spring-summer and when *Synechococcus* was most abundant in winter-early spring. The dominance of clade B phages is not restricted to the Red Sea. Recently, we found that clade B phages were significantly more abundant and infected more cyanobacterial cells than clade A phages in 97% of samples from surface transects across vast regions in the North Pacific Ocean, including samples where *Synechococcus* was more abundant than *Prochlorococcus* by more than 5–10-fold [43]. These patterns are also consistent with metagenomic comparisons of relative read numbers, from both the viral fraction and cellular metagenomes, sampled sporadically from surface waters at various oceanic sites [29, 44].

Intriguingly, clade B phages have significantly higher abundances and infect more cyanobacteria in the environment even though their infection properties show lower fitness than clade A phages. This phenomenon of dominance and more infections by the less virulent virus is not likely to be unique to the T7-like cyanophages, as a slower, less-virulent virus was recently suggested to infect more coccolithophore cells in the Atlantic Ocean [45]. These findings indicate that greater fitness, determined as the greater number of viral progeny produced per unit time in single-host infection settings, does not necessarily predict the dominance of populations in complex communities in the environment.

The dominance of clade B over clade A phages in seasons and in regions with large *Prochlorococcus* populations is likely to be largely due to the ability of many clade B phages but only a minority of clade A phages to infect *Prochlorococcus* [10] (Fig. 5). Since, the dominance of clade B phages was also observed at times (Fig. 4) and in regions [43] where *Synechococcus* was the dominant cyanobacterium, other explanations are required for understanding their high abundances at those times and regions. It is feasible that the greater diversity of clade B phages allows them to infect more *Synechococcus* genotypes than clade A phages. However, it is also possible that the differences in infection properties play a direct role in this phenomenon. These possibilities are not mutually exclusive.

### Modeling abundances based on the infection properties of clade A and clade B phages

Here we address the possibility that the dominance of clade B phages is directly related to their infection properties. This is particularly relevant for when *Synechococcus* is the dominant cyanobacterium since many clade A and clade B phages infect members of this genus. For this, we developed a mathematical model of host-phage population dynamics suitable for narrow host-range phages, in which each phage infects a single susceptible host, and assessed host and phage abundances in steady-state environmental conditions [46] (see Methods). We used the average latent period, burst size and virulence based on our empirical results for clade A and clade B phages and assumed equal decay and contact rates for both phages.

**Fig. 6:**
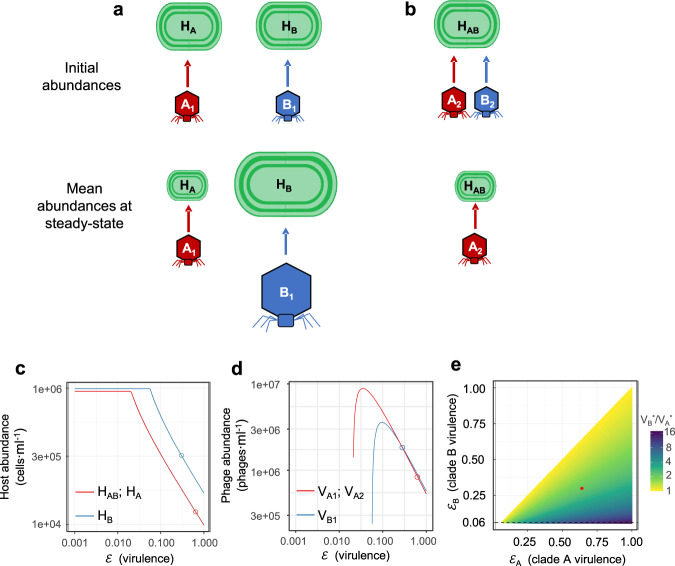
Modeling host and virus population abundances based on infection properties of T7-like cyanophages. Schematic representation of model results of host and virus population sizes at steady-state where clade A and clade B cyanophages infect distinct cyanobacterial genotypes (**a**) or the same cyanobacterial genotype (**b**). Icon areas are approximations of the relative abundance of host and virus populations at steady-state. The change in steady-state abundances of cyanobacterial hosts (**c**) and cyanophages (**d**) as a function of virulence for a clade A (red) and a clade B (blue) cyanophages infecting distinct cyanobacterial genotypes. **d** Cases for clade A and clade B phages are shown when the virulence of the other phage clade (clade B and clade A phages, respectively) were fixed at the averages determined empirically. Open red and blue circles indicate modeled abundances at average virulence values measured in this study for clade A and clade B cyanophages, respectively. **e** Abundance ratios of clade B to clade A cyanophages at steady-state for a range of virulence values in which clade A virulence is greater than clade B virulence. Red closed circle indicates modeled abundance ratio of clade B to clade A cyanophages (2.1) at average virulence values for both clades. The lowest virulence value at which clade B cyanophages persist in the presence of the more virulent clade A cyanophage (0.06) is shown by the dashed horizontal line. See SI Appendix, Table S4 for values of all model parameters, including average virulence for T7-like clade A and clade B cyanophages.

**Fig. 7:**
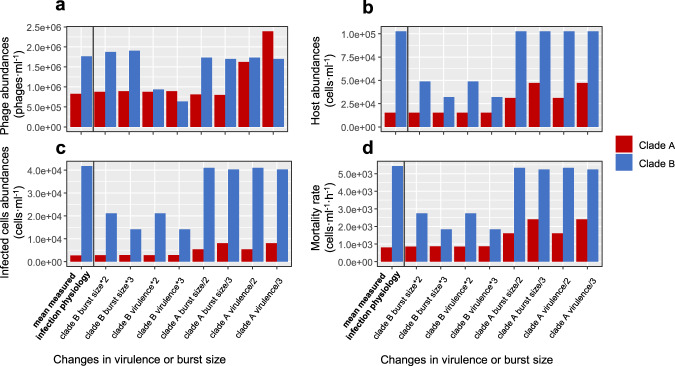
Effect of virulence and burst size on steady-state abundances of cyanobacteria and cyanophages. **a** T7-like cyanophage abundances, **b** host abundances, **c** infected host abundances, **d** mortality rate of hosts, at mean infection physiology levels measured in this study (left most bar in each panel) and as a result of changes in cyanophage burst size and virulence, when infected by clade A or clade B cyanophages. Burst size or virulence values were reduced or increased 2-fold or 3-fold relative to the mean for each clade separately while holding the other variable at the average value for this clade of cyanophages. Values of these variables were held constant at the average for the other phage clade. Latent period was not assessed as this parameter does not influence steady-state abundances of either host or phage in this model (see Eqs. 7, 9, 10 and 12 in Methods). See SI Appendix, Table S4 for values of all model parameters.

We considered highly specific interactions, in which distinct cyanobacterial genotypes (H) were each infected by either a distinct clade A or a distinct clade B phage (V): H_A_ infected by V_A1_ and H_B_ infected by V_B1_. We assumed the same growth rates and carrying capacity for the two hosts. At steady-state, the clade A phage significantly drove down the population size of its host, while the clade B phage reduced its host to a much lesser extent (Figs. 6a, c and 7b). This subsequently resulted in a larger mean population size for the clade B phage relative to the clade A phage (Figs. 6a, d and 7a). Moreover, this model predicts that clade B phages have a greater ecological impact, both infecting more cyanobacteria and causing considerably more cyanobacterial mortality than clade A phages (Fig. 7c, d, SI Appendix, Fig. S5). This is in line with our observations that more *Synechococcus* and *Prochlorococcus* cells are infected by clade B than by clade A cyanophages in the Red Sea (Fig. 5) and in the North Pacific Ocean [43].

Our model indicates that virulence has a strong nonlinear effect on host-phage interactions resulting in non-monotonic outcomes with peak phage abundances, infected cells and virus-induced mortality occurring at intermediate virulence values (Figs. 6d and S5). Towards the lower end of the virulence scale, clade A phages are predicted to be more abundant than clade B phages (Fig. 6d). At higher virulence values, closer to those found empirically in our study, the more virulent clade A phages are less abundant than clade B phages (Figs. 6d, e and 7a), presumably because clade A phages draw down their host populations to such an extent that they do not support large phage populations (Figs. 6c and 7b). In this model formulation, burst size and virulence have equivalent impacts on drawing-down the steady-state host population from the phage-free steady state (Fig. 7b, Eqs. 7 and 10 in Supplementary Methods). However, at infection properties relevant for T7-like cyanophages, virulence has a much stronger effect on phage abundances than does burst size since reduction in virulence of clade A phages would lead to a substantial increase in their abundance (Fig. 7a, Eqs. 9 and 12 in Supplementary Methods).

We also addressed the situation where a distinct clade A phage (V_A2_) and a distinct clade B phage (V_B2_) infect a single host genotype (H_AB_). In this model, the aggressive clade A phage outcompetes the clade B phage (Fig. 6b), as expected due to its superior infection properties (including burst size [46]). When in direct competition, the clade A phage drives down the host population to levels below those that support replication of the clade B phage (see Methods Eqs. 19–23). This competitive exclusion suggests that, in order for clade A and clade B cyanophages to be found in the same body of water, host separation likely occurs spatially or temporally under direct competition, with a particular host genotype being infected by either a clade A or a clade B phage but not both. Indeed, local patches of microbes and interactions on the microscale between microorganisms are likely in planktonic environments [47–49]. We note that multiple phages with similar infection properties could, in principle, infect and coexist on the same host genotype.

Host separation could also result from evolutionary processes. Host evolution through selection for resistance to a phage from either clade would lead to local host separation. The selection pressure for resistance against a clade A phage is likely to be greater than against a clade B phage since resistance to the former would result in the greater increase in host population size. Furthermore, mutations in cyanophages can lead to a change in the hosts they are able to infect [50, 51] and thus allow them to avoid extinction when exposed to direct competition. An example of host separation is apparent in this system for *Prochlorococcus* since many clade B phages can infect *Prochlorococcus* genotypes whereas few clade A phages can. Furthermore, the presence of hundreds of diverse cyanobacterial genotypes [17, 18] with different sensitivities to co-occurring cyanophages [25, 26] in the oceans also supports the possibility of host separation. Similar support for host separation in phage-host interaction networks has also been reported for a variety of other taxa [52–54]. Irrespective of whether host separation is due to ecological and/or evolutionary processes, larger clade B phage populations are predicted to persist when distinct clade A and clade B phage genotypes infect different host genotypes, as described in the first model formulation (Figs. 6a and 7). As such, the lower fitness and virulence of clade B phages can be reconciled with substantially higher abundances of this clade of phages even when *Synechococcus* is the dominant cyanobacterium.

Infection properties may also influence phage population diversity and host range. We hypothesize that intermediate virulence allows clade B phages to infect members of the slower growing *Prochlorococcus* genus [compare [55] and [56]], and perhaps more cyanobacterial types in general under a variety of suboptimal conditions, since clade B phages would reduce their host populations to a lesser extent than the more virulent clade A phages (Figs. 6, 7, SI Appendix, Fig. S6). Having more host types and maintaining larger host populations would result in more overall infections (Fig. 7c). Thus, clade B phages with more overall DNA replication cycles would have greater chances for mutation resulting in increased phage diversity and a greater pool of viral variants available for genetic drift or natural selection. Irrespective of whether the greater diversity of phages and larger repertoire of hosts for clade B phage populations is a consequence of their infection properties or not, the combination of both higher numbers of host types, and intermediate virulence leading to larger sustainable host populations, can explain the greater abundance of clade B T7-like cyanophages over clade A T7-like cyanophages in the ocean.

Our findings raise the possibility that two opposing processes are driving the evolution of virulence in the T7-like cyanophages: Direct phage competition for the same host may lead to the evolution of higher virulence and spatial or temporal host separation. At the community level, however, phage-host separation may select for intermediate virulence which can lead to more sustainable host populations that in turn support larger phage populations. These ideas support the evolution of intermediate virulence in parasites [57, 58], and expand them to include viruses that infect single-celled organisms in complex ecological settings. It will be important for future research to attempt to disentangle the combined effects of multi-scale selection processes [59] in the context of community-level diversity.

## Conclusions

Experimental analyses of phage-host interactions show clear distinctions in infection properties that are delineated with the phylogeny of the two major clades of T7-like cyanophages, with viruses of one clade (clade A) able to infect hosts more rapidly, more productively and with greater virulence than viruses from the other clade (clade B). Yet, as is apparent from our field observations, both clade A and clade B cyanophages persist in nature despite these different infection physiologies. The less aggressive phage clade (clade B) with lower fitness and virulence in single-host infection settings (Fig. 1) is more abundant than the more aggressive phage clade (clade A) over long time scales in nature (Fig. 4), even in waters dominated by the cyanobacterial genus that can be infected by many members of both phage clades. As a result, clade B phages infect more cyanobacterial cells (Fig. 5), and thus have a greater direct ecological impact. These differences between fitness, virulence, diversity and ecological outcome likely arose due to the interdependence of host and virus, where lytic viruses require a host for replication, yet kill off this essential resource during cell lysis and release of progeny viruses. As such, intermediate virulence, smaller burst sizes and relative slowness of infection are likely to be advantageous in preventing rapid collapse of host populations (Figs. 6 and 7). The persistence of less aggressive life history strategies would be especially important for narrow host-range phages, like the T7-like cyanophages, as a substantial reduction of a specific host would result in severe limitation of its key resource. As a result, the evolution of intermediate virulence amongst viruses may help sustain more diverse and larger host populations which, in turn, sustain larger and more diverse virus populations. Overall, our combined experimental and in situ analyses support the role of adaptive evolution in the establishment of discrete cyanophage lineages with significant biological distinctions in cyanophage life history traits, diversity and environmental distribution patterns.

## Methods

### Infection physiology measurements

*Synechococcus* spp. strains WH8109, WH7803, CC9605 were grown in liquid medium, ASW + NO_3_, under a 14/10 h light/dark cycle at a light intensity of 15–20 µmol photons·m^−2^·sec^−1^ and a temperature of 21 °C. The growth rate of these *Synechococcus* strains under these conditions were 0.47, 0.45 and 0.34 d-1 for WH8109, WH7803 and CC9605, respectively (SI Appendix, Table S1). Cyanophages Syn5 and S-TIP37 infect *Synechococcus* WH8109, cyanophages S-CB42 and S-RIP2 infect *Synechococcus* WH7803 and cyanophages S-TIP28 and S-TIP67 infect *Synechococcus* CC9605 (Table 1). Phages Syn5, S-CB42 and S-TIP28 belong to clade A and phages S-TIP37, S-RIP2 and S-TIP67 belong to clade B. Cultures were infected at mid-log phase of growth (at ~5 × 10^7^ to 10^8^ cells·ml^−1^) and at an MOI of 2. Infection dynamics (adsorption kinetics and latent period) were determined in viral growth curve experiments whereby the number of free infective cyanophages in the extracellular medium was determined with time after phage addition by plaque assay. The same host was used for the experiments and the plaque assay.

Virulence and burst size were measured using a single cell approach [30]. Cells from infected cultures were sorted by flow cytometry, at the time of maximum adsorption for each phage (SI Appendix, Table S2) into 96-well plates. At an MOI of 2, all cells are expected to have come into contact with a phage prior to maximal adsorption. For virulence assays, cells were sorted into culture-containing wells and incubated under host growth conditions for up to two weeks. Visual clearing of the wells relative to uninfected control plates was used to determine the percent lysis. For burst size assays, cells were sorted into medium-containing wells and incubated overnight to allow for lysis. The number of infective phages produced per cell was determined by plating each well on a separate Petri dish using the plaque assay.

Cyanophage decay rates were determined by measuring the loss of infective phages from freshly produced lysates. Lysates were incubated in glass tubes under host growth conditions (see above) at a light intensity of 20 μmol photons∙m^−2^ ∙ s^−1^. The loss of infectivity was quantified using the plaque assay. See Supplementary Methods for more details on the methodology.

### Field sampling

Sampling was carried out at Station A (29°28'N, 34°55'E), 180 km north of the Straits of Tiran in the Gulf of Aqaba, Red Sea, above a bottom depth of 720 m. Samples for nearly all measurements were collected monthly between March 2013 and February 2014, except for infected cyanobacteria samples which were collected in March and September 2014. Samples were collected using a rosette with 11 L Niskin Go-Flo bottles (General Oceanic) on the *RV Sam Rothberg* during National Monitoring Program (NMP) cruises. Samples were collected every 20 m from the surface to 140 m as well as from 200 m and 400 m depths.

Water column conditions, including temperature, salinity and pressure, were measured in-situ by a CTD instrument (SBE 19plus V2 SeaCAT Profiler). Macronutrients (total oxidized nitrogen (NO_3_^−^ + NO_2_^−^) and phosphate (PO_4_^3−^) were determined using a QuickChem 8000 flow injection analyzer (Lachat Instruments) [60]. Chlorophyll *a* was extracted using cold acetone (90%) and measured with a Turner TD700 fluorometer [60]. Abundances of *Synechococcus*, *Prochlorococcus*, eukaryotic phytoplankton and heterotrophic bacteria were analyzed with a LSR-II flow cytometer (BD Biosciences) equipped with a 488 nm laser. *Synechococcus* and *Prochlorococcus* were identified based on their phycoerythrin and chlorophyll autoflorescence, respectively, as well as from forward scatter which is a proxy for size [38]. Data for all of the above measurements were obtained from the NPM database (http://www.meteo-tech.co.il/EilatYam_data/ey_data.asp). See Supplementary Methods for more details.

### Virus measurements

Samples for virus measurements were prefiltered through a 20 µm mesh. For free virus quantification, VLPs and T7-like cyanophages, samples were then filtered over a 0.2 µm syringe filter (Millex-GV 33 mm 0.22 µm PVDF). VLP samples were fixed in 1.6% formaldehyde for 20 min in the dark, frozen and stored at −80 °C. Samples were filtered onto 0.02 µm Anodisc aluminum oxide filters (Whatman, Kent, UK), stained with SYBR Green I and visualized and enumerated by epifluorescence microscopy [61].

Samples for quantification of T7-like cyanophage were frozen and stored at −80 °C (without fixation) and quantified using the polony method [37]. In this solid phase, single molecule PCR method the DNA polymerase gene from T7-like cyanophages is amplified using degenerate primers. Amplicons resulting from clade A and clade B cyanophages are hybridized with clade-specific probes and visualized with a GenePix 4000B microarray scanner (Axon Instruments).

The percent infection of cyanobacteria by clade A and clade B T7-like cyanophages was done using the iPolony method [38]. Cells (after 20 µm prefiltration) were fixed in 0.1% glutaraldehyde in the dark for 30 min, flash frozen in liquid nitrogen and stored at −80 °C until analysis. Sorting was performed on a BD Influx flow cytometer equipped with a 488 nm laser and small particle detector on one-drop purity mode. Thousands of sorted *Synechococcus* and *Prochlorococcus* cells from each sample were screened for the presence of intracellular T7-like cyanophage DNA using primers that detect the DNA polymerase gene. Supplementary Methods for more details.

### Statistical analysis

Contour plots were created in Ocean Data View (R Schlitzer, http://odv.awi.de/) with weighted-average gridding. Determination of normal distribution was based on Kolmogorov-Smirnov and Shapiro-Wilk tests. *T*-test with Equality of Means was used for comparison of normally distributed groups. Otherwise, the nonparametric Mann-Whitney test was used. All tests were performed using the ‘stats’ package in R (R core team, 2013). The correlation between *Prochlorococcus* and cyanophages was analyzed using a nonparametric Spearman’s test.

### Host-virus population modeling

To model how differences in infection properties between clade A and clade B T7-like cyanophages translate into population abundances in the environment, we used the results of our infection experiments in a mathematical model of phage-bacteria population dynamics [46]. The mathematical model includes host cell division, viral infection, viral-induced lysis of host cells and the release of virions, and extracellular decay of virus particles. This core population model was modified to include the probability of successful infection based on measured virulence data and a virus loss term due to contact with uninfected hosts. This includes contacts that do not result in infection as we assume that all contacts result in adsorption but not that all adsorptions result in successful infection. The infected cells are lysed at a rate proportional to the inverse of the latent period [46].

In the first model we investigated the dynamics of host-virus interactions when one host genotype (*H*_*A*_) is infected by a clade A phage (*V*_*A*_) and another host genotype (*H*_*B*_) is infected by a clade B phage (*V*_*B*_) (Fig. 6a). The second model considers a situation whereby a single cyanobacterial genotype (*H*_*AB*_) is infected by both a clade A (*V*_*A*_) and a clade B (*V*_*B*_) phage (Fig. 6b). We assume phages have burst sizes (*β*), virulence (*φ*), and latent periods (1/*η*) that are equal to the mean values measured empirically in this study for the clade A and clade B phages. Contact rate (*φ*) and decay coefficients (*m*) were assumed to be equal for both phage clades and are based on literature values. Note that we use these population dynamic models to understand the qualitative relationship between infection physiology of cyanophages and their ecological impacts, rather than for detailed time series reconstruction of host-virus abundances and population dynamics. See Table S4 for definitions of all model parameters and their initial values and Supplementary Methods for a description of the models. The code for these models is available at https://github.com/lindelllab/Maidanik-et-al-2021.git.

## Supplementary information

Supplementary Information

## Data Availability

The data generated during this study are included in this published article and its Supplementary Information files or are available from the corresponding author upon reasonable request.
