# Supplementary material for: Cyanophages from a less virulent clade dominate over their sister clade in global oceans
Source: ISME J. 2022 Jun 20;16(9):2169–80. doi: 10.1038/s41396-022-01259-y (PMC9381782; doi:10.1038/s41396-022-01259-y)
Supplement: Supplementary file 1 — Supplementary Information [file 41396_2022_1259_MOESM1_ESM.pdf]

Supplementary Information

Cyanophages from a less virulent clade dominate over their sister clade in global oceans

Ilia Maidanik[1], Shay Kirzner[1], Irena Pekarski[1], Laure Arsenieff[1], Ran Tahan[1], Michael C. G. Carlson[1], Dror Shitrit[1], Nava Baran[1], Svetlana Goldin[1], Joshua S. Weitz[2,3,4], Debbie Lindell[1*]

[1]Faculty of Biology, Technion – Israel Institute of Technology, Haifa 3200003, Israel

[2]School of Biological Sciences, Georgia Institute of Technology, Atlanta, GA 30332, USA

[3]School of Physics, Georgia Institute of Technology, Atlanta, GA 30332, USA

[4]Institut de Biologie, École Normale Supérieure, Paris, 75014, France

*Corresponding author: dlindell@technion.ac.il

Table of Contents:

Supplementary Methods ………………………………………………………………... 3
Supplementary References …………………………………………………………... 12
Table S1: Latent periods of T7-like cyanophages ………………………………… 14
Table S2: Time of maximal adsorption and sorting for burst size and virulence assays....... 15
Table S3: Plaque sizes …………………………………………………………….…... 15
Table S4: Model variable and parameter values ……………………………..……... 16
Fig. S1: The annual dynamics of eukaryotic phytoplankton and heterotrophic bacteria at Station A in the Gulf of Aqaba, Red Sea. …………………………................ 17
Fig. S2: The annual dynamics of T7-like cyanophages relative to virus-like particle abundances at Station A in the Gulf of Aqaba, Red Sea. ………………………… 18
Fig. S3: Depth profiles of clade A and clade B T7-like cyanophages over the annual cycle. ……………………………………………………………………. 19
Fig. S4: Correlation of clade A T7-like cyanophage to *Synechococcus* in the photic zone (0-140 m) in four representative depth profiles. …………………………….. 20
Fig. S5: Modeling infection and mortality rates of cyanobacteria based on infection properties of T7-like cyanophages. ……………………………………………….. 21
Fig. S6: Effect of model variables and values on host and virus population dynamics. ….. 22

Supplementary Methods

Cyanobacteria growth measurements

Synechococcus growth was measured by chlorophyll *a* autofluorescence of the cells using a Turner 10AU field fluorometer at 340-500 nm excitation and >665 nm emission or a SynergyMx plate reader at 440±20 nm excitation and 680±20 nm emission. Determination of *Synechococcus* concentrations in cultures was done by flow cytometry using the LSR-II (BD) for cultures of WH8109 and CC9605 or with the ImageStreamX (Amnis, Seattle, WA) for WH7803 cultures since the latter strain formed aggregates in 33% of the cases (75 out of 225 cells investigated in 4 different experiments). The number of aggregates and the number of cells per aggregate were taken into consideration when determining the per cell burst size and virulence for WH7803 (see below). Flow cytometry data were collected and analyzed as described below for field samples. ImageStreamX data were analyzed using the IDEAS6.2 software.

Virus growth curve experiments and adsorption kinetics

Virus growth curve experiments [62] were used to assess the dynamics of the infection cycle. The concentration of phages in the extracellular medium is determined with time after phage addition to the host culture. The time point with the minimum concentration of free viruses is considered the time of maximal adsorption. The time point prior to the release of new phage progeny into the extracellular medium is considered the end of the latent period.

Cultures were infected with cyanophages at the mid-log growth phase of hosts (~$5x10^7$ to 10^8 cells·ml^{-1}) at an MOI of 2. Samples were collected at various time points, diluted 100-fold in ASW+NO_3 medium [63, 64] and filtered through 0.2 µm pore-sized Acrodisc Syringe Filters (Millex-GV 33 mm 0.22 µm PVDF). The filtrate was serially diluted and cyanophages enumerated using the plaque assay (see below). Three to eight independent experiments were performed for each host-phage interaction.

Adsorption kinetics of viruses to host cells were calculated by fitting a linear regression to the ln transformed phage abundances versus time for the period between phage addition and maximal adsorption, with the slope being the rate of adsorption. Due to the rapid adsorption of S-TIP28, its adsorption kinetics was determined from the difference of the ln transformed initial abundances and abundances at maximal adsorption divided by time. Data for these calculations were obtained from the growth curve experiments shown in Fig. 1 except for the Syn5 phage. Adsorption kinetics for Syn5 was calculated from specific adsorption experiments in which samples were collected every 3 min at an MOI of 0.01.

Comparison of phage growth curves was done using a multi-level modeling approach [65, 66], in which we tested the combined effect of the type of the virus and time after infection with random effect of the replicate cultures. The model was performed using the 'lme4' package [67], the p-values were calculated using the 'broom.mixed' package [68]. The figures were produced using the 'ggplot2' package [69].

Plaque assay and plaque size estimate

The plaque assay was used to quantify the number of infective viruses in a lysate or experimental sample. Serial dilutions of the lysate were mixed with the assay culture and combined with 0.28% ultraPure low melting point (LMP) agarose (Invitrogen) in ASW+NO_3 medium and poured into petri dishes with 1 mM sodium sulfite [25, 55].

Plaque sizes were compared for each pair of clade A and clade B phages after growth on lawns of the same host cultures for a period of 4 or 5 days (*SI Appendix,* Table S3). Petri dishes were photographed and the diameter of the plaques were measured using the ImageJ Java image processing program (https://imagej.nih.gov/ij/).

Virulence and burst size measurements

Virulence and burst size was evaluated using a single cell approach [30]. Cells at 5-10x10^7 cells·ml^{-1} were infected at an MOI of 2 so that all cells would contact an infective phage. At maximum adsorption, cells were sorted by flow cytometry into individual wells in 96-well plates in PBS (137 mM NaCl, 2.7 mm KCl, 10 mM Na_2HPO_4, 1.8 mM KH_2PO_4 at pH 7.4; Dulbecco's Phosphate Buffered Saline, Biological Industries) using the FACSAria-IIIu (BD) cell sorter with an 85 µm nozzle at a pressure of 45 psi.

For the virulence assay, cells were sorted into culture-containing 96-well plates and incubated under growth conditions for up to two weeks. If the sorted cell lyses, this initiates infection and lysis of the culture in the well. The percentage of wells with a significant decrease in cyanobacterial color relative to uninfected control plates was used to score the number of lysed cells in the original sorted culture. Results are shown as % of lysed cells for phages infecting WH8109 and CC9605, and as % lysed cells per sorted event for phages infecting WH7803 since 33% of cells produced aggregates of more than 1 cell (see above). Six to 21 experiments were carried out for each host-phage interaction, with each experiment consisting of 120 cells sorted into internal wells of two 96-well plates.

For the burst size, single cells were sorted into individual wells of medium-containing 96-well plates. The plates were incubated in host growth conditions overnight, a period longer than the length of the lytic cycle, to allow lysis. The number of viruses produced by each cell was determined by plating the contents of each well on separate petri dishes using the plaque assay. Plates with single plaques were excluded from the analysis since it is not possible to differentiate between cases where a cell produced a single plaque or lysis was delayed and we plated prior to cell lysis. The results of the assay are shown as the number of viruses produced per lysed cell. Since 33% of the WH7803 cells formed aggregates, we calculated the burst size for phages on this strain by dividing the number of phages produced per sorted event by 2, the combined average number of cells in over 50 sorted events per experiment, including singlets and aggregates.

Decay measurements

The decay rate was determined for freshly lysates. Triplicate cultures were infected at a MOI of ~0.1 and phages were allowed to replicate until the host culture declined, but prior to complete lysis of the cultures. Lysates were collected after filtering the cultures through 0.2 µm PVDF filters and were incubated in glass tubes under host growth conditions: a 14/10 h light/dark cycle at a light intensity of 20 µmol photons·m^{-2}·s^{-1} and a temperature of 21ºC. The loss of infectivity was quantified over the period of a month by the plaque assay. Decay rates were calculated by fitting linear regressions to the ln transformed plaque abundances versus time with the slope of the regression giving the rate of decay.

Field study site

The Gulf of Aqaba is the northwest basin of the Red Sea. The deepest point reaches 1800 m. On average the Gulf of Aqaba is 14 km wide and is an evaporation basin surrounded by deserts, with almost no entry of water runoff. The only influx of surface water is through the Straits of Tiran, which are characterized by a shallow sill at a depth of 252 m. The water temperature fluctuates from ~27 °C at the surface during summer to 20.5 °C in winter, the latter of which is the water temperature below the stratified layer throughout the year. The lack of cold deep water results in annual deep winter mixing [20, 35, 70]. Winter mixing is followed by temperature-induced stratification, formed by a combination of increased solar radiation and pulses of warm advected water that increases the temperature in the upper 100-150 m by 1-2°C in a matter of days [70].

Physical environmental conditions

Water column conditions including temperature, salinity and pressure, were measured in-situ by the CTD instrument (SBE 19plus V2 SeaCAT Profiler). The σ density anomaly was calculated using the Ocean Data View software algorithm [71]. Here we define the maximal mixing depth as the depth down to which a uniform distribution of σ density was observed, with a difference of less than 0.01 kg·m^{-3} per meter. We present environmental data from the beginning of the calendar year in 2013 to provide information on water column conditions during mixing prior to the beginning of our sampling period in the spring.

Discrete water sampling

Sample collection for annual analyses began in March 2013 and continued monthly until February 2014. In addition, samples were collected for percent infection in March and September of 2014. The samples were collected on the cruises of the National Monitoring Program (NMP) at sampling Station A (29°28'N, 34°55'E) using a rosette with 11 L Niskin Go-Flo bottles (General Oceanic) on the *RV Sam Rothberg*. Samples were collected from nine depths in two depth profiles: 80, 100, 140, 200 and 400 m generally at ~08:00 AM Coordinated Universal Time (UTC) and 0, 20, 40 and 60 m at ~09:30 AM (UTC) which was from 10:00-12:30 local time. Samples for chlorophyll *a* and flow cytometry counts were filtered through a 60 µm plankton mesh while samples for virus enumeration were prefiltered through a 20 µm mesh into darkened bottles and kept at room temperature while being processed for the different procedures (see below).

Macronutrients and chlorophyll a *concentrations*

Samples for analysis of macronutrient, total oxidized nitrogen (TON: $NO_3^- + NO_2^-$) and phosphate (PO_4^{3-}), were collected without prefiltration, into 12 ml NUNC tubes (Thermo-Fisher Scientific) and were kept at 4ºC in the dark until analysis with a QuickChem 8000 flow injection analyzer (Lachat Instruments) [60]. The detection limits of TON and PO_4^{3-} are 0.05 µM and 0.03 µM, respectively.

Chlorophyll *a* concentrations were determined using a cold acetone extraction procedure [60]. Water (300 ml) was filtered onto a GF/F filter (Whatman GF/F, 25 mm), placed in 90% acetone and incubated for 24 h at 4°C in the dark. Extracted chlorophyll *a* concentrations were determined using a non-acidification fluorometric method with a pre-calibrated Turner TD700 fluorometer.

Enumeration of photosynthetic cells and bacteria

Samples for determining photosynthetic cells (*Synechococcus*, *Prochlorococcus,* eukaryotic phytoplankton) and bacterial abundances were fixed in 0.125% glutaraldehyde, frozen in liquid nitrogen and stored at -80°C until analysis. The samples were analyzed with the LSR-II flow cytometer (BD Biosciences) equipped with a 488 nm laser from 200 µl subsamples in two or three technical replicates. As an internal reference for size and fluorescence intensity, 1 µm diameter yellow-green microspheres (Fluoresbrite) were added. The data from the LSR-II was analyzed using FCSexpress 5.

Abundances of *Synechococcus*, *Prochlorococcus,* and photosynthetic picoeukarya were determined based on their characteristic autofluorescence and size, determined from forward scatter. *Prochlorococcus* and photosynthetic picoeukaryotic cells were detected by red fluorescence of chlorophyll *a* (emission at 692/640nm) while *Synechococcus* was detected by orange fluorescence of phycoerythrin (emission at 580/30nm) [72]. The photosynthetic picoeukaryotes and *Prochlorococcus* were distinguished from each other by their difference in forward scatter and the intensity of their red fluorescence. Quantification of *Prochlorococcus* is underestimated in the upper 40 m during stratification due to their very low chlorophyll *a* fluorescence and insufficient sensitivity of the LSR-II for their detection. Due to this underestimation of *Prochlorococcus* we show correlation results for depths from 60 to 140 m in the main text. We note here that a significant correlation was also found when the 0 to 40 m samples were included and was $\rho=0.74$ ($S=28362$, $p<2.2e-16$, $n=76$) for the annual comparison.

Enumeration of total bacteria was done after DNA staining with SYBR Green I (Invitrogen) at a dilution of 10^{-4} of the commercial stock solution [72] and a 15 min dark incubation. Detection of heterotrophic prokaryotes was based on the green fluorescence of SYBR Green I, excited with the 488 nm laser and detected by a 530/20 nm filter. Heterotrophic bacteria were quantified as the difference between the abundances of total bacteria and cyanobacteria. Therefore, during the stratification period they will be overestimated by the same degree that *Prochlorococcus* is underestimated.

Quantification of virus-like-particles (VLPs)
Bulk virioplankton was determined from VLP abundances following Patel [61]. Samples were filtered on-board over a 0.2 μm syringe filter (Millex-GV 33 mm 0.22 μm PVDF) into two 50 ml falcon tubes, fixed at a final concentration of 1.6% formaldehyde (Bio Lab) (that had been 0.02 μm filtered). Samples were incubated for 20 minutes in the dark and frozen and stored until analysis at -80°C. Samples were thawed at 25°C and 2-4 ml was immediately filtered onto 0.02 μm Anodisc aluminum oxide filters (Whatman, Kent, UK), stained with SYBR Green I (Molecular Probes Inc., Eugene, OR, USA), and enumerated by epifluorescence microscopy with a Cell Observer microscope (Zeiss) equipped with an AxioCam HS video camera, filters for blue excitation (470/40nm) and green emission (525/50nm) a 1000X magnification. Signals with a radius of 3-12 pixels were considered virus-like-particles, based on a comparison with manual counts. Images of 20 fields from each sample were analyzed using OpenCFU 3.9.0 software [73].

Quantification of T7-like cyanophages
The quantification of T7-like cyanophages was done using the polony method as described previously [37]. Samples collected from Niskin bottles were filtered over a 0.2 μm syringe filter as above. The <0.2 μm filtrate, containing the viral fraction, was frozen in liquid nitrogen after 24 hours and stored at -80°C until analysis. In the lab 8.3 μl of the <0.2 μm filtrate was added to 27.5 μl gel mix containing 10% acrylamide, 20 μM acrydite primer Acr-534-Rd, 0.2% BSA, 0.1% APS, 0.1% TEMED and poured into a well in custom-made microscope slides (Thermo Fisher Scientific) and polymerized in an argon chamber for 30 min. The acrydite primer is used to anchor the amplicon to the acrylamide gel. Degenerate primers targeting the DNA polymerase gene were used to amplify T7-like cyanophages at the genus level: forward primer 341Fd-15-NNN: NNNCCNAAYYTNGSNCAR, and reverse primer Acr-534Rd: [5Acr]TGNWRYTCRTCRTGNAYRAA. The final volume of the gel on the slide is 11.6 μl. The slides were washed with MilliQ and 0.025% Tween-20 to remove the excess gel and dried in air for 20-30 minutes.

After polymerization the other PCR reagents were diffused into the gel in a 20 μl volume. These are 1X PCR buffer for Jumpstart *Taq* polymerase (Sigma-Aldrich), 0.25mM dNTPs, 10 μM unmodified primer 341-Fd-15-NNN, 0.1% Tween-20, 0.2% BSA, 13.4 units of Jumpstart *Taq* polymerase (Sigma-Aldrich)). The slides were sealed with a Secure-Seal hybridization chamber (Grace Biolabs) and filled with mineral oil. Thermocycling was carried out in a twin-tower slide thermocycler (DNA Engine with dual block slide chamber, Bio-Rad). Prior to cycling, an initial denaturation step at 94°C for 5 min is used to make the viral DNA in the capsids accessible. A total of 50 cycles are performed with denaturation at 94°C for 45 s, annealing at 50°C for 45 s and elongation at 72°C for 2 min. A final 6 min elongation step was performed at 72°C after which the slides were stored at 10 °C overnight.

After PCR slides were washed in hexane for 2-5 minutes and twice in wash buffer E (10 mM Tris (pH 7.5), 50 mM KCl, 2 mM EDTA (pH 8.0), 0.01% Triton X-100). Denaturation of the double-

stranded polonies was done in 70% formamide with 1X SSC buffer (150 mM NaCl, 15 mM sodium citrate) for 15 min at 70ºC followed by washes in milliQ water and wash buffer E to remove the unbound DNA strand. Differential detection of the clade A and B T7-like cyanophages was done by hybridization with clade-specific probes that carry different fluorescent modifications; Cy3 and Cy5 for clade A and B, respectively. The hybridization mix contained 6X SSPE (900 mM NaCl, 90 mM NaH_2PO_4, 6 mM EDTA), 0.1% Triton X-100 and the fluorescently labeled probes: 0.45µM of Cy5-405 BF (d+3i) probe (TAYGCITTYYTITAYGGIGC) and 0.15µM of Cy5-478BF (d+3i)_tip42 probe (TAYTGITTYYTITAYGGIGG) for clade B phages; and 1.2µM of Cy3-405 AF (d+3i) probe (TAYTGYYTIATITAYGGIGG) for clade A phages. The hybridized polonies were identified using a GenePix 4000B microarray scanner (Axon Instruments). Cy5 and Cy3 labeled probes were detected using the 635 and 532 nm lasers, respectively. Polony enumeration was done manually using the ImageJ Java image processing program (https://imagej.nih.gov/ij/).

Reliable quantification is achieved with at least 10 polonies per gel with a limit of accurate quantification of 1×10^4 phages·ml^{-1}. When fewer polonies are observed, sample concentration is required for accurate quantification. A 40-50-fold concentration for clade A phage quantification was done for 4 representative depth profiles using iron flocculation [37, 74], decreasing the lower threshold of accurate quantification to 200 phages·ml^{-1}. $FeCl_3$ was added to the samples at a final concentration of 0.18 mM and incubated at room temperature for at least 1 h for samples collected in April, July and August 2013 and January 2014. After incubation the samples were centrifuged for 10 min at 17,000Xg at 10°C and then resuspended in a buffer containing 0.125 M Tris, 0.1 M Na_2EDTA and 0.125 M oxalic acid, pH 6.

Quantification of cyanobacterial infection by T7-like cyanophages

The quantification of percent infection by clade A and clade B T7-like cyanophages was done using the iPolony method [38]. Samples collected from Niskin bottles were filtered through a 20 µm Nylon mesh. Cells in the filtrate were fixed with 0.1% glutaraldehyde (final concentration) in the dark for 30 minutes and then frozen in liquid nitrogen and stored at -80°C until analysis. Sorting was performed on a BD Influx flow cytometer equipped with a 488 nm laser and small particle detector on one-drop purity mode. *Synechococcus* and *Prochlorococcus* were gated based on their autofluorescence properties and size as described above. Thousands of sorted *Synechococcus* and *Prochlorococcus* cells from each sample were screened for the presence of intracellular T7-like cyanophage DNA using the polony method as described above. Percent infection was calculated by dividing the number of cyanophage amplicons by the number of cells input in each reaction. Corrections for co-sorted free cyanophages and differential detection across the latent period were then applied as described previously [38].

Host-virus population modeling

In our first model we investigated the dynamics of host-virus interactions when one host genotype (H_A) is infected by a clade A phage (V_A) and another host genotype (H_B) is infected by a clade B phage (V_B) (Fig. 6a). Phages have burst sizes (β), virulence (ε), and latent periods ($1/\eta$) that are

equal to the mean measured in this study for the clade A and clade B phages. For clade A these are β_A = 103.8 phage·cell^{-1}, ε_A = 0.64, 1/η_A = 3.3 h, while for clade B these are β_B = 35 phage·cell^{-1}, ε_B = 0.30, 1/η_B = 7.7 h (Table S4). Contact rate (φ) and decay coefficient (m) were assumed to be equal for both phage clades and are based on literature values. The contact rate was 10^{-7} ml·virus^{-1}·h^{-1} assuming 1 µm and 40 nm diameter for cyanobacteria and T7-like cyanophage, respectively [75] and a decay coefficient of 0.02 h^{-1} was used [76]. The carrying capacity *(K)* was set to 10^6 cells·ml^{-1}, a common maximal abundance of cyanobacteria in subtropical waters [20, 77] and a growth rate (r) of 0.06 h^{-1} was used for the cyanobacteria based on *Synechococcus* [78, 79]. See Table S4 for definitions of all parameters and their initial values.

(1) $$\frac{dH_A}{dt} = \underbrace{rH_A\left(1-\frac{N}{K}\right)}_{\text{cyanobacteria growth}} - \underbrace{\varepsilon_A\varphi V_A H_A}_{\text{loss due to infection}}$$ where $N = H_A + H_B$

(2) $$\frac{dI_A}{dt} = \underbrace{\varepsilon_A\varphi V_A H_A}_{\text{newly}} - \underbrace{\eta_A I_A}_{\text{lysed cells}}$$

(3) $$\frac{dV_A}{dt} = \underbrace{\beta_A\eta_A I_A}_{\text{viral production}} - \underbrace{mV_A}_{\text{viral loss due to decay}} - \underbrace{\varphi V_A H_A}_{\text{viral loss due to adsorption}}$$

(4) $$\frac{dH_B}{dt} = rH_B\left(1-\frac{N}{K}\right) - \varepsilon_B\varphi V_B H_B$$

(5) $$\frac{dI_B}{dt} = \varepsilon_B\varphi V_B H_B - \eta_B I_B$$

(6) $$\frac{dV_B}{dt} = \beta_B\eta_B I_B - mV_B - \varphi V_B H_B$$

In Eqs. 1 and 4 the term $rH\left(1-\frac{N}{K}\right)$ denotes the growth of cyanobacterium (H) limited by the environmental carrying capacity of the host (K) where r is the cyanobacterial growth and N is the total abundance of uninfected cells. The model assumes that additional cyanobacterial loss is only due to viral infection, $\varepsilon\varphi VH$, controlled by the interaction between hosts and viruses and the probability of successful infection (assuming that the bulk of contacts are with susceptible hosts).

Eqs. 2, and 5 describe the dynamics of cells infected by clade A and clade B phages. The term $\varepsilon\varphi V$H is the production of newly infected cells and ηI is the product of the number of infected cells, I, lysed at rates η. As modeled here, the length of an infection is exponentially distributed with mean infection periods of $1/\eta$, respectively, where β is the burst size.

Eqs. 3 and 6 describe the dynamics of the free phage. The term $\eta I\beta$ describe the production of new phages due to the lysis of infected cells where β is the burst size and mV is the loss of phage due to decay. φVH is the combined loss of viruses due to decay and infection of a new host. The terms φVH are the loss of phage due to adsorption to uninfected cells.

The solution of the equations at steady-state for both host-virus interactions are:

(7) $H_A{}^* = \frac{m}{(\mathcal{E}_A\beta_A-1)\varphi}$

(8) $I_A{}^* = \frac{mr}{\eta_A(\mathcal{E}_A\beta_A-1)\varphi}\ (1-\frac{N^*}{K})$

(9) $V_A{}^* = \frac{r}{\mathcal{E}_A\varphi}\ (1-\frac{N^*}{K})$

(10) $H_B{}^* = \frac{m}{(\mathcal{E}_B\beta_B-1)\varphi}$

(11) $I_B{}^* = \frac{mr}{\eta_B(\mathcal{E}_B\beta_B-1)\varphi}\ (1-\frac{N^*}{K})$

(12) $V_B{}^* = \frac{r}{\mathcal{E}_B\varphi}\ (1-\frac{N^*}{K})$

The ratio of clade A and clade B abundances was determined from Eqs. 9 and 12, while the relative infection levels were calculated from Eq. 8 and Eq. 11. We also used our model to predict the mortality rates of cyanobacteria caused by clade A versus clade B phages. At steady-state, the growth of the cyanobacterial host is balanced by the mortality caused by viral lysis. Consequently, the ratio of total mortality rates is the ratio of the steady-state host densities H_B^*/H_A^* , as shown below in Eq 13, because the same growth rate and carrying capacity is assumed for cyanobacterial hosts of both clade A and clade B phages.

(13) $\frac{rH_B^*\left(1-\frac{N}{K}\right)}{rH_A^*\left(1-\frac{N}{K}\right)} = \frac{H_B^*}{H_A^*}$

The second model experiment considers a situation whereby a single cyanobacterial genotype (H_{AB}) is infected by both a clade A (V_A) and a clade B (V_B) phage (Fig. 6b).

(14) $\frac{dH_{AB}}{dt} = rH_{AB}\left(1 - \frac{H_{AB}}{K}\right) - \varepsilon_A \varphi V_A H_{AB} - \varepsilon_B \varphi V_B H_{AB}$

(15) $\frac{dI_A}{dt} = \varepsilon_A \varphi V_A H_{AB} - \eta_A I_A$

(16) $\frac{dI_B}{dt} = \varepsilon_B \varphi V_B H_{AB} - \eta_B I_B$

(17) $\frac{dV_A}{dt} = \beta_A \eta_A I_A - mV_A - \varphi V_A H_{AB}$

(18) $\frac{dV_B}{dt} = \beta_B \eta_B I_B - mV_B - \varphi V_B H_{AB}$

The solution of the equations, for $\varepsilon_A \beta_A > \varepsilon_B \beta_B$, at steady-state for both host-virus interactions are:

(19) $H_{AB}^* = \frac{m}{(\varepsilon_A \beta_A - 1)\varphi}$

(20) $I_A^* = \frac{mr}{\eta_A(\varepsilon_A \beta_A - 1)\varphi}\ (1 - \frac{H_{AB}^*}{K})$

(21) $V_A^* = \frac{r}{\varepsilon_A \varphi}\ (1 - \frac{H_{AB}^*}{K})$

(22) $I_B^* = 0$

(23) $V_B^* = 0$

Our models are necessarily simplifications of the complexity of interactions found in nature [58]. They do not explicitly account for the influence of other mortality agents on dynamics, such as grazing and viral lysis via other cyanophage families including those with broad host-ranges, which would stabilize population oscillations around steady state solutions (Fig. S6). Other factors, such as the rate of phage decay, host growth rates, and carrying capacity also influence steady-state outcomes and oscillations of these interactions (Fig. S6). As such, we use these population dynamic models to understand the directionality of the relationship between infection physiology of cyanophages and their ecological impacts. All numerical solutions to the differential equations were calculated using the deSolve [80]. The code for these models is available at https://github.com/lindelllab/Maidanik-et-al-2021.git.

References

62. Burleson FG, Chambers TM, Wiedbrauk DL. Viral replication: One-step growth curve. *Virology*. 1992. Elsevier, pp 100–106.

63. Wyman M, Gregory RP. F, Carr NG. Novel role for phycoerythrin in a marine cyanobacterium, *Synechococcus* strain DC2. *Science* 1985; **230**: 818–820.

64. Lindell D, Padan E, Post AF. Regulation of *ntcA* expression and nitrite uptake in the marine *Synechococcus* sp. strain WH7803. *J Bacteriol* 1998; **180**: 1878–1886.

65. Dobson AJ, Barnett A. An introduction to generalized linear models. 2008. Chapman and Hall/CRC.

66. Muthén B, Asparouhov T. Growth mixture modeling: Analysis with non-Gaussian random effects. *Longitud Data Anal* 2008; **143165**.

67. Bates D, Mächler M, Bolker B, Walker S. Fitting linear mixed-effects models using lme4. *J Stat Softw* 2015; **67**: 1-48.

68. Robinson D, Hayes A. broom: Convert statistical analysis objects into tidy tibbles. 2019. *https://broom.tidymodels.org/*

69. Wickham H. tidyverse: Easily install and load the "Tidyverse". *J Open Source Softw* 2017; **4:** 1686.

70. Carlson DF, Fredj E, Gildor H. The annual cycle of vertical mixing and restratification in the Northern Gulf of Eilat/Aqaba (Red Sea) based on high temporal and vertical resolution observations. *Deep Sea Research Part I: Oceanogr Res Pap* 2014; **84**: 1–17.

71. Schlitzer R. Ocean Data View. *https://odv.awi.de.*

72. Marie D, Brussaard CPD, Thyrhaug R, Bratbak G, Vaulot D, Marie D, et al. Enumeration of marine viruses in culture and natural samples by flow cytometry enumeration of marine viruses in culture and natural samples by flow cytometry. *Appl Environ Microbiol* 1999; **65**: 45–52.

73. Geissmann Q. OpenCFU , a new free and open-source software to count cell colonies and other circular objects. *PLoS One* 2013; **8**: 1–10.

74. John SG, Mendez CB, Deng L, Poulos B, Kauffman AKM, Kern S, et al. A simple and efficient method for concentration of ocean viruses by chemical flocculation. *Environ Microbiol Rep* 2011; **3**: 195–202.

75. Talmy D, Beckett SJ, Taniguchi DAA, Brussaard CPD, Weitz JS, Follows MJ. An empirical model of carbon flow through marine viruses and microzooplankton grazers. *Environ Microbiol* 2019; **21**: 2171–2181.

76. Suttle CA, Chen F. Mechanisms and rates of decay of marine viruses in seawater. *Appl Environ Microbiol* 1992; **58**: 3721–9.

77. Malmstrom RR, Coe A, Kettler GC, Martiny AC, Frias-Lopez J, Zinser ER, et al. Temporal dynamics of *Prochlorococcus* ecotypes in the Atlantic and Pacific oceans. *ISME J* 2010; **4**: 1252–64.

78. Agawin NSR, Duarte CM, Agustí S. Growth and abundance of *Synechococcus* sp. in a Mediterranean Bay: Seasonality and relationship with temperature. *Mar Ecol Progr Ser* 1998; **170**: 45–53.

79. Liu H, Campbell L, Landry M. Growth and mortality rates of *Prochlorococcus* and *Synechococcus* measured with a selective inhibitor technique. *Mar Ecol Progr Ser* 1995; **116**: 277–287.

80. Soetaert K, Petzoldt T, Setzer RW. Solving Differential Equations in R : Package deSolve. *J Stat Softw* 2010; **33**: 1–25.

81. Wang K, Chen F. Prevalence of highly host-specific cyanophages in the estuarine environment. *Environ Microbiol* 2008; **10**: 300–12.

Table S1: Latent periods of T7-like cyanophages

Cyanophage	Clade /subclade[#]	Host strain	Host growth rate (d^{-1})	Phage latent priod (h)	Source of virus isolates
S-CBP42	A/4D	*Synechococcus* WH7803	0.45	3 h n=4 (this study)	Chesapeake Bay, Jun-06 [81]
S-CBP2	A/[&]	*Synechococcus* CB0208	N/A	8-10 h [81]	Chesapeake Bay, Sep-02 [81]
P60	A/2D	*Synechococcus* WH7805	N/A	1.5 h [81]	Satilla River ,Jul-88 [81]
Syn5	A/1D	*Synechococcus* WH8109	0.47	1 h ([28], this study)	Sargasso Sea, Jul-90 [41]
S-TIP28	A/3D	*Synechococcus* CC9605	0.34	2 h n=4 (this study)	Gulf of Aqaba, Red Sea, Sep-07 [14]
S-RIP2	B/5D	*Synechococcus* WH7803	0.45	8 h n=6 (this study)	Rhode Island Sound, Oct-07 [14]
S-TIP37	B/5D	*Synechococcus* WH8109	0.47	3 h n=3 (this study)	Gulf of Aqaba, Red Sea, May-09 [14]
S-TIP67	B/1D	*Synechococcus* CC9605	0.34	8 h n=4 (this study)	Gulf of Aqaba, Red Sea, Mar-09 [14]
S-CBP1	B/11D	*Synechococcus* CB0101	1.1 [75]	6-8 h [81]	Chesapeake Bay, Jul-02 [81]
S-CBP3	B/8D	*Synechococcus* CB0101	1.1	8-10 h [81]	Chesapeake Bay, Jul-04 [81]
P-SSP7	B/14D	*Prochlorococcus* MED4	0.35-0.6	7-8 h [27] (this study)	Sargasso Sea, Sep-99 [15]
P-GSP1	B/14D	*Prochlorococcus* MED4	0.35	8-10 h n=4 (this study)	Gulf Stream Jul-00 [15]
P-TIP2	B/12D	*Prochlorococcus* MED4	0.35	6-8 h n=2 (this study)	Gulf of Aqaba, Red Sea, Nov-07 [25]
P-SSP5	B/9D	*Prochlorococcus* MIT9515	0.35	8-10 h n=2 (this study)	Sargasso Sea, Sep-99 [19]
P-RSP1	B/9D	*Prochlorococcus* MIT9215	0.35	8-12 h n=2 (this study)	Gulf of Aqaba, Red Sea, Jul-00 [15]

[#]Subclade designations based on Dekel-Bird et al. (2013) and Sun et al. (2018). [&]Not placed into a discrete subclade. N/A – no report of host growth rate for conditions used to determine the latent period.

Table S2: Time of maximal adsorption and sorting for burst size and virulence assays

Cyanophage	Clade	Host strain	Latent priod (h)	Maximal adsorption/sorting time (hours after phage addition)
S-CBP42	A	*Synechococcus* WH7803	3 h	2 h
Syn5	A	*Synechococcus* WH8109	1 h	0.5 h
S-TIP28	A	*Synechococcus* CC9605	2 h	0.5 h
S-RIP2	B	*Synechococcus* WH7803	8 h	5 h
S-TIP37	B	*Synechococcus* WH8109	3 h	2 h
S-TIP67	B	*Synechococcus* CC9605	8 h	4 h

Table S3: Plaque sizes

Host	Phage	Days after infection	n*	Plaque diameter (mm)
WH8109	Syn5	4	31	16.1±3.0
	S-TIP37	4	12	6.8±2.0
WH7803	S-CBP42	5	13	12.9±1.3
	S-RIP2	5	39	3.0±0.5
CC9605	S-TIP28	4	12	15.6±2.0
	S-TIP67	4	56	3.8±0.6

*Number of plaques measured

Table S4: Model variable and parameters values

Symbol	Description	Value of parameters
H_A	Abundance of susceptible host for infection by clade A	
H_B	Abundance of susceptible host for infection by clade B	
H	Abundance of susceptible host for infection by both clades	
V_A	Clade A virus abundance	
V_B	Clade B virus abundance	
r	Host growth rate	0.06 h^{-1}
φ	Contact rate	10^{-7} $ml \cdot viruses^{-1} \cdot h^{-1}$
m	Decay coefficient	0.1 h^{-1}
K	Carrying capacity	10^6 $cell \cdot ml^{-1}$
ε_A	Probability of cell lysis after adsorption of clade A virus	0.64*
ε_B	Probability of cell lysis after adsorption of clade B virus	0.30*
β_A	Clade A burst size	103.8 $phages \cdot cell^{-1}$ *
β_B	Clade B burst size	35 $phages \cdot cell^{-1}$ *
$1/\eta_A$	Clade A latent period	3.3 h**
$1/\eta_B$	Clade B latent period	7.7 h**

* Average values of three interactions for both clade A and clade B (Table 1)
** Average values of 5 clade A phages and 10 clade B phages (Table S1)

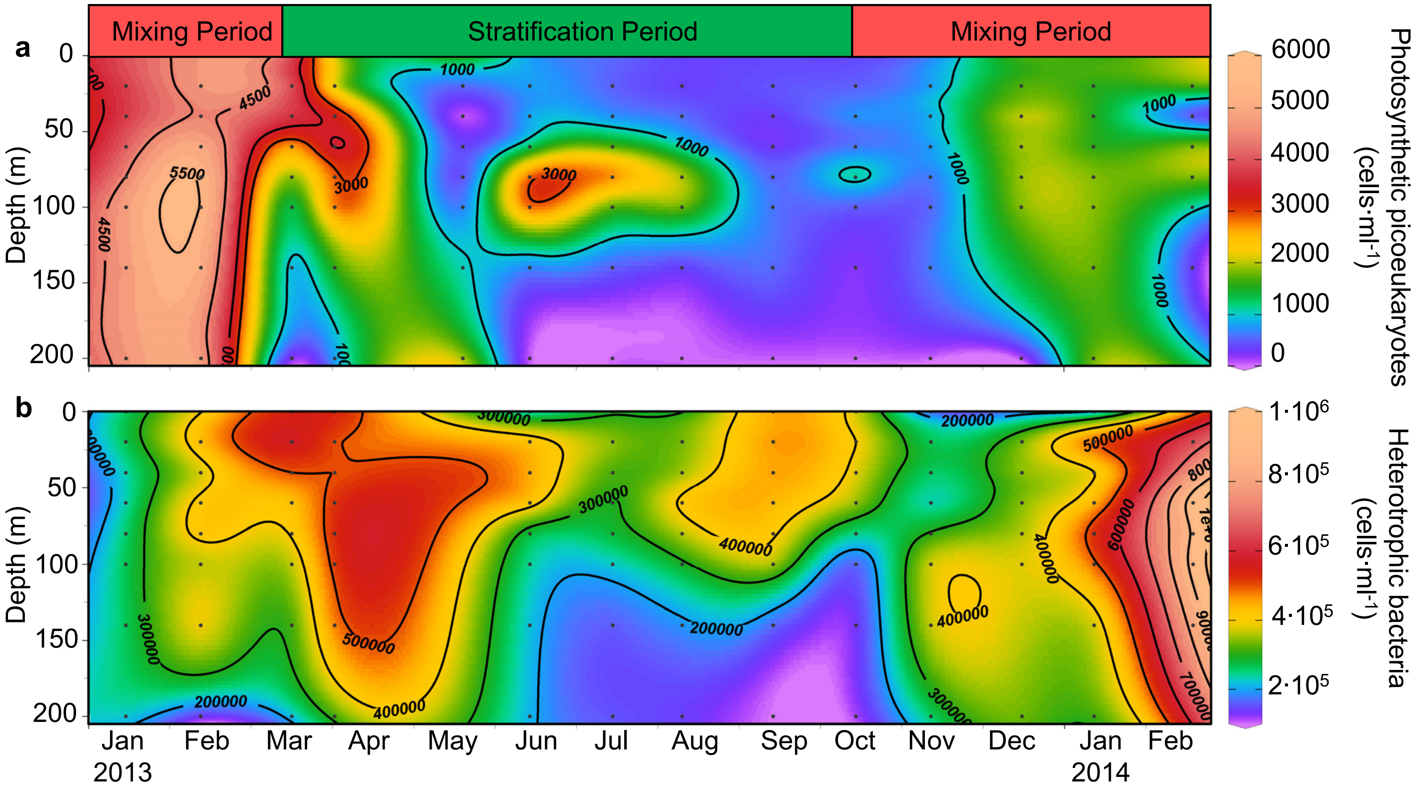

Fig. S1: **The annual dynamics of eukaryotic phytoplankton and heterotrophic bacteria at Station A in the Gulf of Aqaba, Red Sea. a,** Picoeukaryote phytoplankton, **b**, heterotrophic prokaryotes. The black points indicate actual samples. The contour plots are interpolations performed using DIVA gridding. The color bar above the plots indicates the mixing and stratification periods. All of the plots were created by Ocean Data View (http://odv.awi.de/).

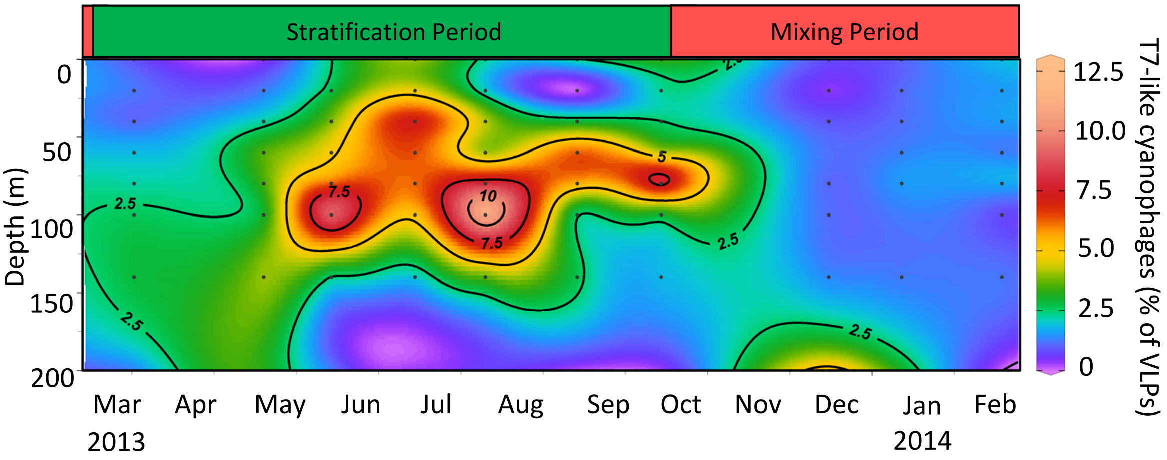

Fig. S2: **The annual dynamics of T7-like cyanophages relative to virus-like particle abundances at Station A in the Gulf of Aqaba, Red Sea.** The black points indicate actual samples. The contour plots are interpolations performed using DIVA gridding. The color bar above the plots indicates the mixing and stratification periods. The plot was created by Ocean Data View (http://odv.awi.de/).

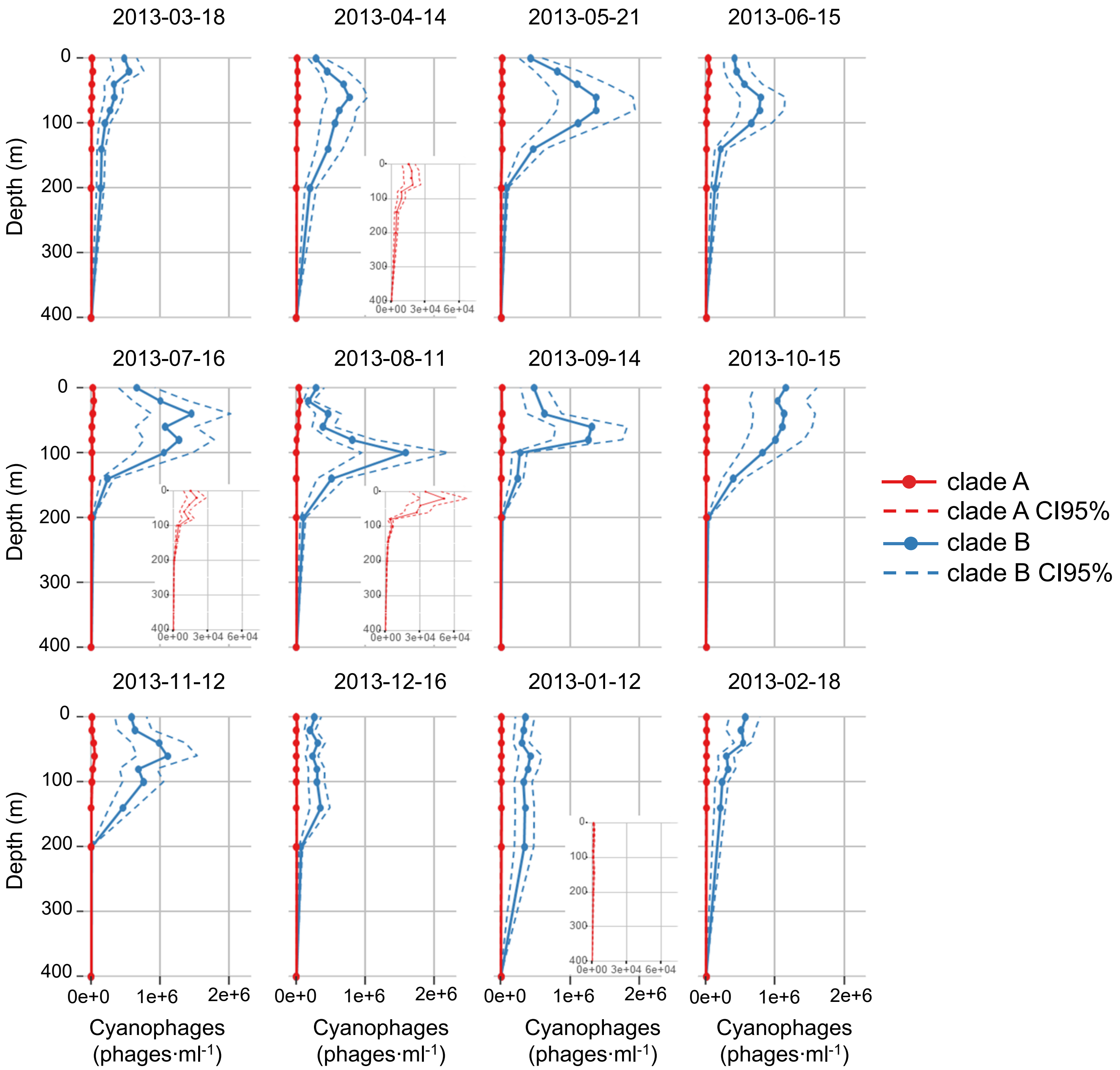

Fig. S3: **Depth profiles of clade A and clade B T7-like cyanophages over the annual cycle.** Solid red and blue lines show the abundances of clade A and clade B cyanophages, respectively, as determined from polony experiments. The dashed lines indicate 95% confidence intervals. The inset graphs show quantification after iron chloride concentration for clade A cyanophages. The date of sampling is shown above each panel (year-month-day).

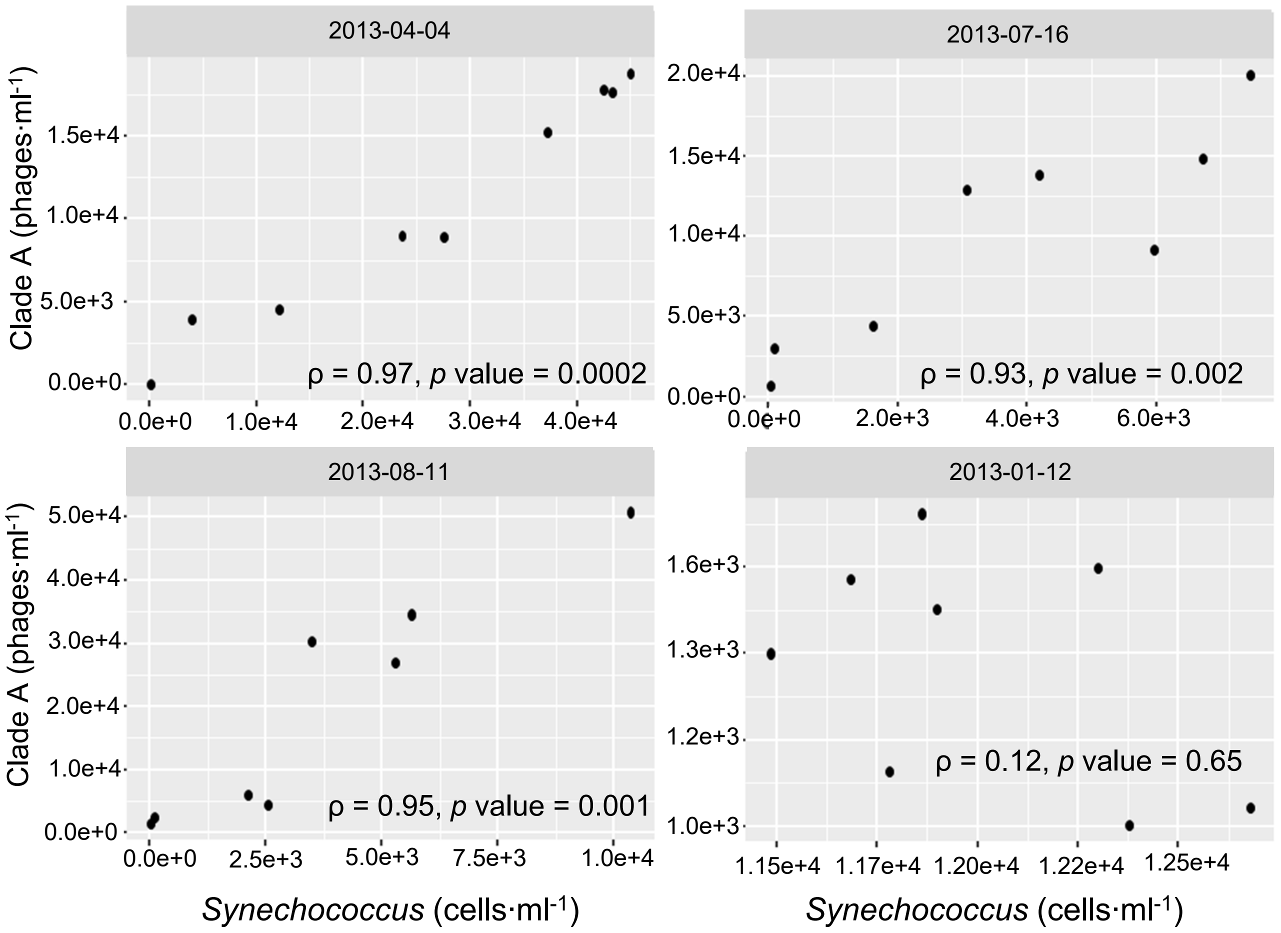

Fig. S4: Correlation of clade A T7-like cyanophage to *Synechococcus* in the photic zone (0-140 m) in four representative depth profiles. The abundance of clade A phages were analyzed by the polony method after iron chloride concentration. *Synechococcus* abundances were analyzed by flow cytometry. The date of sampling is shown above each panel (year-month-day).

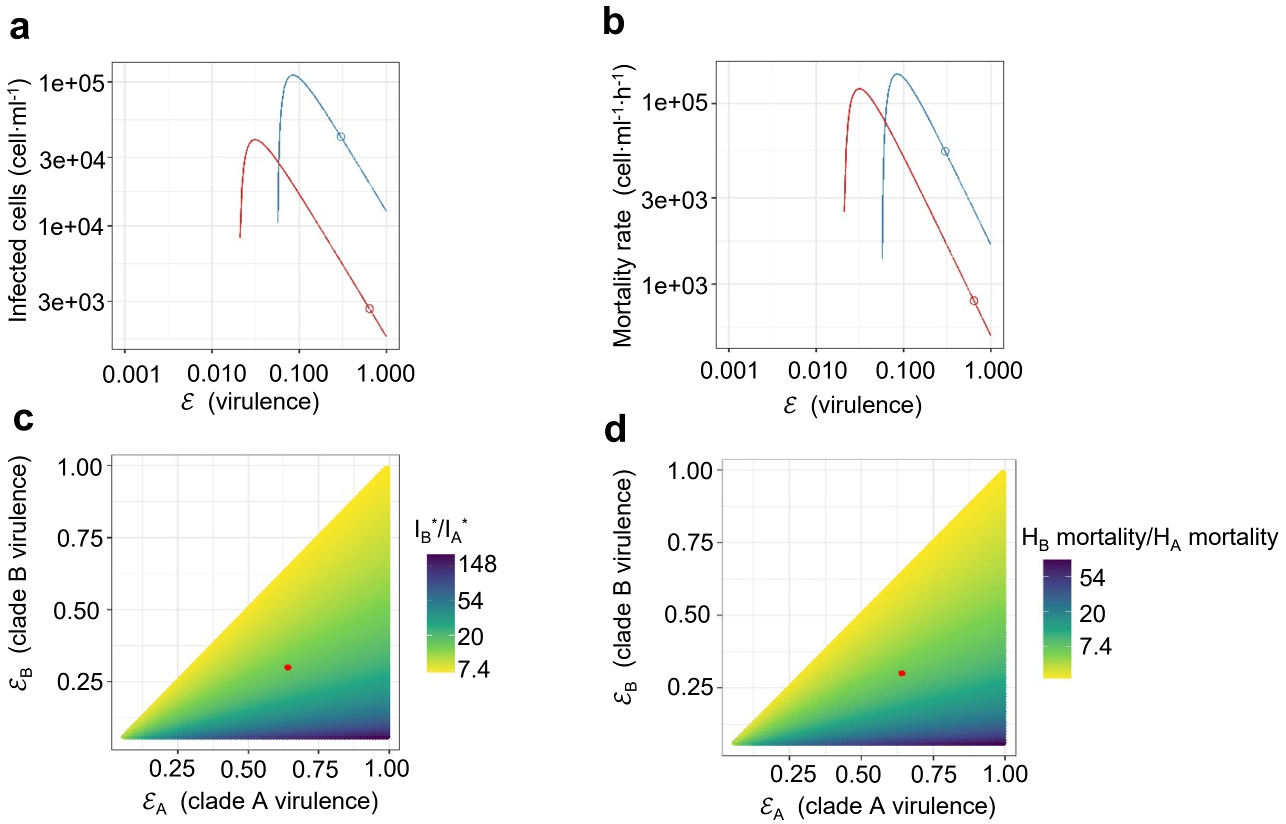

Fig. S5. Modeling infection and mortality rates of cyanobacteria based on infection properties of T7-like cyanophages. a, Changes in steady-state abundances of infected cyanobacterial hosts, and **b***,* changes in mortality rates, as a function of virulence for a clade A (red) and a clade B (blue) cyanophage infecting distinct cyanobacterial genotypes. For **a** and **b** we show the cases for clade A and clade B when the virulence of the other clade (clade B and clade A phages, respectively), were fixed at the average determined empirically in this study. Open red and blues circles (**a-b**) indicate model results at the average virulence values measured in this study for clade A and clade B cyanophages, respectively. **c**, Relative abundances of infected cells, and **d***,* relative mortality rates of cyanobacteria at steady state shown for the range of virulence values in which the virulence of clade A is greater than for clade B. Clade B phages are predicted to result in more infected cells and cause considerably more mortality than clade A phages for all cases where the virulence of the clade B phage is lower than that of the clade A phage. Closed red circles (**c-d**) indicate ratios of model results at the average virulence values, with 15.7-fold more infected cells (**c**) and 6.7-fold more mortality (**d**) by clade B than by clade A cyanophages.

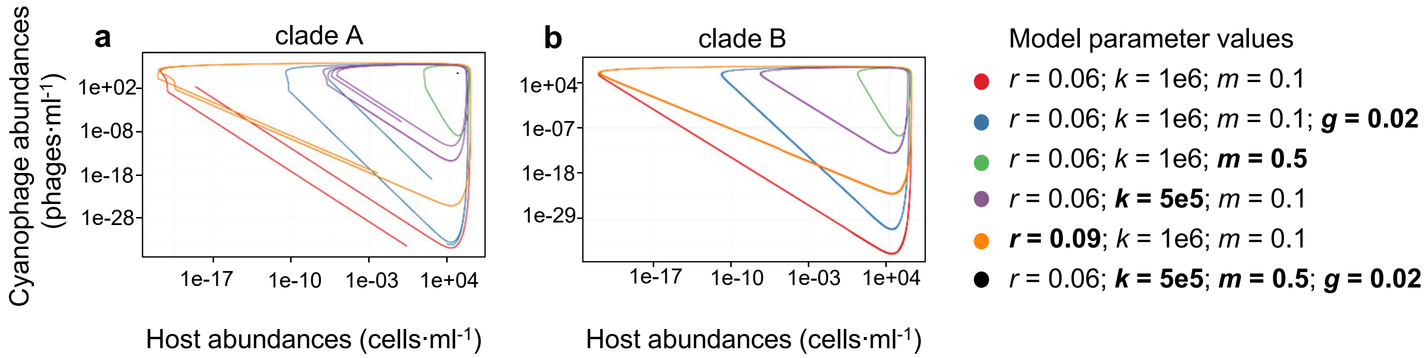

Fig. S6: Effect of model variables and values on host and virus population dynamics. Phase plane diagram of oscillations of free phages and uninfected hosts prior to reaching steady-state for clade A (**a**) and clade B (**b**) T7-like cyanophages when different or additional model parameter values were considered: host growth rate, *r*; host carrying capacity, *k*; viral decay rate, *m*; and grazing rate, *g*. Host and phage dynamics with the parameter values used in model simulations (main text and Fig. 6) are shown in red (see *SI Appendix,* Table S4). The values changed or parameter added relative to this are shown in bold in the legend. Dynamics oscillate less with increased host growth rate (h^{-1}), reduced host carrying capacity (cells ml^{-1}), increased phage decay rates (h^{-1}) and when the grazing rate (h^{-1}) is included. Note that a clade B phage with parameter values of r = 0.06; k = 5e5; m = 0.5; g = 0.02 declines to become extinct and is not shown. The addition of the grazing terms of *gH* (grazing of susceptible hosts) and *gI* (grazing of infected hosts) changes Eq. 1, 2, 4 and 5 (see Methods) to:

(1) $\frac{dH_A}{dt} = rH_A\left(1-\frac{N}{K}\right) - \varepsilon_A\varphi_A V_A H_A - gH_A$ where $N = H_A + H_B$

(2) $\frac{dI_A}{dt} = \varepsilon_A\varphi V_A H_A - \eta_A I_A - gI_A$

(3) $\frac{dH_B}{dt} = rH_B\left(1-\frac{N}{K}\right) - \varepsilon_B\varphi_B V_B H_B - gH_B$

(4) $\frac{dI_B}{dt} = \varepsilon_B\varphi V_B H_B - \eta_B I_B - gI_B$
